# Various LncRNA Mechanisms in Gene Regulation Involving miRNAs or RNA-Binding Proteins in Non-Small-Cell Lung Cancer: Main Signaling Pathways and Networks

**DOI:** 10.3390/ijms241713617

**Published:** 2023-09-03

**Authors:** Eleonora A. Braga, Marina V. Fridman, Alexey M. Burdennyy, Vitaly I. Loginov, Alexey A. Dmitriev, Irina V. Pronina, Sergey G. Morozov

**Affiliations:** 1 Institute of General Pathology and Pathophysiology, 125315 Moscow, Russia; burdennyy@gmail.com (A.M.B.); loginov7w@gmail.com (V.I.L.); zolly_sten@mail.ru (I.V.P.); sergey_moroz@list.ru (S.G.M.); 2 Research Centre for Medical Genetics, 115522 Moscow, Russia; 3 Vavilov Institute of General Genetics, Russian Academy of Sciences, 119991 Moscow, Russia; marina-free@mail.ru; 4 Engelhardt Institute of Molecular Biology, Russian Academy of Sciences, 119991 Moscow, Russia; alex_245@mail.ru

**Keywords:** lncRNA, mRNA, miRNA, ceRNA model, RNA-binding protein, hnRNP

## Abstract

Long non-coding RNAs (lncRNAs) are crucial players in the pathogenesis of non-small-cell lung cancer (NSCLC). A competing binding of lncRNAs and mRNAs with microRNAs (miRNAs) is one of the most common mechanisms of gene regulation by lncRNAs in NSCLC, which has been extensively researched in the last two decades. However, alternative mechanisms that do not depend on miRNAs have also been reported. Among them, the most intriguing mechanism is mediated by RNA-binding proteins (RBPs) such as IGF2BP1/2/3, YTHDF1, HuR, and FBL, which increase the stability of target mRNAs. IGF2BP2 and YTHDF1 may also be involved in m^6^A modification of lncRNAs or target mRNAs. Some lncRNAs, such as DLGAP1-AS2, MALAT1, MNX1-AS1, and SNHG12, are involved in several mechanisms depending on the target: lncRNA/miRNA/mRNA interactome and through RBP. The target protein sets selected here were then analyzed using the DAVID database to identify the pathways overrepresented by KEGG, Wikipathways, and the Reactome pathway. Using the STRING website, we assessed interactions between the target proteins and built networks. Our analysis revealed that the JAK-STAT and Hippo signaling pathways, cytokine pathways, the VEGFA-VEGFR2 pathway, mechanisms of cell cycle regulation, and neovascularization are the most relevant to the effect of lncRNA on NSCLC.

## 1. Introduction

Lung cancer is a malignant neoplasm of epithelial origin characterized by a severe course and high mortality. Lung cancer is divided into two large groups based on histological characteristics: small-cell lung cancer (SCLC) and non-small-cell lung cancer (NSCLC). This review discusses NSCLC, which accounts for more than 85% of all lung cancers. According to the modern classification of NSCLC, the majority of cases (at least 40%) comprise adenocarcinomas, followed by cases of squamous cell carcinomas, and a fraction of large-cell lung cancer [1,2].

It is important to bear in mind that an oncological disease stems not only from genetic aberrations caused by changes in DNA structure but also from impaired epigenetic processes associated with dysregulation of gene expression and gene interactions in signaling pathways. Indeed, many epigenetic factors may trigger NSCLC. In particular, current research focuses on the role of non-coding RNAs, the so-called “dark matter” of genome. According to two large-scale genome projects, ENCODE and FANTOM, protein-coding genes account for no more than 2–3% of the human genome, while 80% of the human genome is transcribed but stays mostly untranslated [3,4].

RNAs that do not encode proteins (ncRNAs) are currently considered critical regulators in cancer [5,6]. There are different classes of these molecules, with each class being unique and characterized by its own pool of mechanisms and regulations. Recently, two largest classes, microRNAs (miRNAs) and long non-coding RNAs (lncRNAs), have gained the greatest importance in understanding the functional significance of regulating interactions between different levels of cellular mechanisms for preserving homeostasis as well as aberrant interactions of the same processes in tumor cells [7].

MiRNAs are endogenous ~23-nt RNAs that direct post-transcriptional regulation via pairing with mRNAs of protein-coding genes. The presence of complementary regions in miRNAs and 3′UTR sequences of mRNAs (miRNA response elements, or MREs) ensures the competitive interaction of different mRNAs with miRNAs, the so-called mechanism of competing endogenous RNAs, ceRNAs [8,9]. In this case, a single miRNA can regulate the expression of many mRNAs, and a single mRNA can be regulated by multiple miRNAs. The regulation of miRNAs is mediated by a complex set of proteins termed the RNA-induced silencing complex. It has been established that miRNAs control the expression of more than 50% of protein-coding genes. This means that they regulate many vital processes of cellular activity: proliferation, differentiation, apoptosis, adhesion, epithelial–mesenchymal transition (EMT), and metastasis, as confirmed by studies in tumors of various origins [10]. The discovery of miRNA and the ceRNA mechanism marked a revolutionary step in molecular biology and genetics. To date, other mechanisms of the influence of miRNAs on the expression of protein genes through the regulation of transcription, alternative splicing, and translation have also been explored [11]. The ENCODE consortium placed miRNAs at the very core of regulatory networks, assigning them the role of “master regulators” of signaling cascades in the cell [12].

LncRNAs are a class of non-coding RNAs more than 200 nucleotides long that do not produce protein products because there is no open reading frame or they are not long enough. They act as important regulators of many cellular processes [13]. The number of lncRNAs identified in humans exceeds 60,000 [14]. LncRNAs can possess the properties of tumor suppressors, oncogenes, and lncRNAs with dual functions which can be explained, in particular, by different effects/phenotypes of the same target mRNA in different cell types or conditions [15,16].

The most studied mechanism of gene regulation involving lncRNAs in the last decade is the miRNA-mediated interaction between lncRNAs and mRNAs, which reflects the lncRNA/miRNA/mRNA axis. Studying ceRNA involvement requires identifying the common binding sites (MREs) for miRNAs in both mRNA and lncRNA sequences [17]. Discovering lncRNAs and establishing the role of ceRNA in their regulatory functions has become a new breakthrough in modern biology.

Studying ceRNA networks in various cancer types has shown that ceRNA-related molecules have a more conserved structure and may play key roles in both normal and carcinogenic processes. Currently, a substantial number of studies present various lncRNA/miRNA/mRNA interactomes that affect NSCLC development and progression. Several reviews published in the past few years have discussed the function and mechanism of action of lncRNAs via the ceRNA model in NSCLC [1,7,18].

Recently, the mechanism of regulation of cancer-related genes involving a combination of lncRNA with RNA-binding proteins (RBPs) also attracted the attention of researchers. RBPs combine a flexible structure with a versatile RNA-binding domain [19,20,21] which allow RBPs to enter into highly dynamic interactions with both proteins and coding and non-coding RNAs, resulting in the formation of the ribonucleoprotein complexes (RNPs) [20,21,22]. Therefore, RBPs can regulate mRNA expression and stability at the post-transcriptional level [20,21]. We found about 20 studies on the role of the interactions of lncRNAs and RBPs in the post-transcriptional regulation of cancer-related genes in NSCLC. There are also a number of studies that analyze the direct effect of lncRNA on target mRNA.

In our review, we discuss various mechanisms of gene regulation involving lncRNAs in NSCLC: the mechanism mediated by miRNA-dependent competing endogenous RNAs and alternative models comprising the RBP model and the model involving the direct effect of lncRNAs on mRNAs. It is worth noting that we have not found any reviews summarizing studies on the role of the combination of lncRNAs with RBPs in the regulation of tumor genes, neither in NSCLC nor in any other cancer (PubMed, accessed on 10 June 2023).

Of note, the papers were selected for analysis relying primarily on the evidence-based methods employed by the authors. Current research includes bioinformatic prediction and screening of potential experimental targets using the data on differential expression and presence of complementary regions. Here, we mostly summarized experimental studies using a robust set of methods, including clinical specimen work, cell culture, transfection with mimetics and silencers, Western blotting, and in vivo animal models, besides bioinformatic prediction. Furthermore, we predominantly selected studies that provided evidence in favor of direct binding of lncRNA to miRNA, lncRNA to mRNA, or RBP, proved using specific techniques such as RNA immunoprecipitation (RIP), dual-luciferase reporter assays, or RNA pull-down experiments.

A deep understanding of these mechanisms based on non-covalent binding of lncRNA to mRNA, DNA, miRNA, and RNA-binding proteins, in which steric complementarities play an important role, is impossible without an analysis of 3D structures. Although such studies have not yet been reported for NSCLC, the methods of 3D analysis and structural modeling are developing, and it was shown that folding and maintaining the structure seem to be important for encoding lncRNA functions and interactions [23,24].

In the last section, the protein sets selected in our comprehensive review are analyzed using the DAVID database to identify pathways overrepresented by KEGG, Wikipathways, and the Reactome pathway. Moreover, proteins regulated by lncRNAs through ceRNA and alternative mechanisms are considered separately. To filter out the most common functions of regulatory lncRNAs, we assessed the interactions between target proteins and built networks using the STRING website.

In summary, here we attempt to overview the regulatory role of lncRNAs in the pathogenesis of NSCLC on comprehensive and large-scale data.

## 2. Mechanism of Competing Endogenous LncRNAs in Gene Expression Regulation in NSCLC

The first explored regulatory RNAs to be scrutinized were miRNAs. The mechanism of miRNAs’ action on protein-coding mRNAs has been investigated for several decades [8,9] and is relatively well understood. The most studied and accepted mechanism of lncRNAs’ involvement in gene regulation is competition of lncRNAs with mRNAs for binding to miRNAs. This is the so-called competing endogenous RNA (ceRNA) model which involves lncRNAs absorbing miRNAs like a sponge, preventing their interaction with target mRNAs [2,17]. Based on this model, it is possible to compile multiple axes of interactions between lncRNAs, miRNAs, and mRNAs.

### 2.1. Potential lncRNA/miRNA/mRNA Interactome Axes

LncRNA/miRNA/mRNA axes directed by oncogenic lncRNAs are presented in Table 1.

Let us discuss the examples of studies performed using a variety of different methods. For example, the role of lncRNA MFI2-AS1 in the development of NSCLC was analyzed using determination of the associated lncRNAs, miRNAs, and mRNAs in online databases [70]. Subsequently, the sponge-mediated interaction was confirmed using a dual luciferase test and RNA immunoprecipitation (RIP) assays, which confirmed the binding of a significant fraction of lncRNA to the Argonaute2 (Ago2) protein. The axis MFI2-AS1/miR-107/NFAT5 was confirmed to trigger proliferation, angiogenesis, migration, and other processes in lung cells, leading to their malignization and NSCLC progression (Table 1). These effects were demonstrated in the experiments on cell lines using preliminary inactivation of MFI2-AS1, followed by treatment with exosomes containing MFI2-AS1. In contrast, simultaneous treatment with exosomes lacking MFI2-AS1 and silencing of this lncRNA in the tumor cell lines suppressed proliferation and migration. Thus, the authors suggested an important role MFI2-AS1 played in NSCLC development [70]. The study on RNA exosomes secreted by tumor tissues appears to be promising, as the exosomes can affect both the tumor and its microenvironment.

Classical isolation of RNA with a subsequent real-time PCR was applied to evaluate the expression level of MEG8 in cancer and adjacent normal tissues [69]. The results showed a significant increase in the expression level in the tumor compared with the adjacent tissues. Furthermore, these findings were confirmed in cancer cell lines. Transfection of cancer cells demonstrated MEG8 overexpression and a simultaneous downregulation of the MEG8 target-miR-107. The dual luciferase gene reporter method used to verify the relationship identified the competing endogenous mRNA CDK6 (Table 1). The crucial role of the axis MEG8/miR-107/CDK6 in proliferation, cell cycle changes, invasion, and migration that stimulate cancer progression was confirmed by various direct methods including flow cytometry and a cell scratch test. Finally, the MEG8/miR-107/CDK6 axis was demonstrated to regulate NSCLC tumor progression through activating an Rb/E2F3 pathway.

Studying the pathways involved in the regulation of apoptosis, which is crucial for NSCLC development, revealed an axis regulated by MINCR lncRNA [71]. It was further confirmed by in vitro experiments on cell lines as well as clinical samples of NSCLC. The study utilized various methods such as qRT-PCR, Western blotting, plasmid construction, and assays for colony formation and apoptosis, which showed the correlation of MINCR upregulation and poor prognosis for NSCLC patients. The study also revealed that the downregulation of this lncRNA resulted in cell proliferation and migration inhibition and promoted apoptosis. According to dual luciferase reporter assay, miR-126 targeted this lncRNA. In this study, SLC7A5 was identified as a direct target of miR-126, and the MINCR/miR-126/SLC7A5 interactome was confirmed as a regulator of NSCLC progression through apoptosis inactivation (Table 1).

Another scientific group studying apoptosis regulation showed that overexpression of MIR9-3HG lncRNA upregulated the LIMK1 mRNA in NSCLC, which was decreased by miR-138-5p [72] (Table 1). Epithelial–mesenchymal transition (EMT) is known as a crucial process in cancer metastasis. Apoptosis inhibition is another mechanism of metastasis development. Evaluating the regulatory role of MIR9-3HG in various intracellular processes required an arsenal of experimental methods, such as qRT-PCR, Western blotting, and luciferase reporter, to validate that MIR9-3HG lncRNA regulates cell proliferation, migration, invasion, and EMT in NSCLC [72]. Therefore, these findings can prove useful for NSCLC therapy following further investigations.

As shown in Table 1, approximately 80 interactomes have been identified for oncogenic lncRNAs, thus implicating the contribution of lncRNA in the processes of invasion, EMT, and metastasis of NSCLC. Numerous studies revealed that the oncogenic lncRNAs play also a role in drug resistance development.

Importantly, the studies based solely on bioinformatics approaches are less convincing than studies harnessing both predictive and experimental methods, including the analysis of clinical samples, cell culture, transfection with mimetics and silencers, Western blotting, and animal studies in vivo. Moreover, in most of the cited works, direct binding of microRNAs to both lncRNA and mRNA has been confirmed by various methods such as RNA immunoprecipitation (RIP), dual-luciferase reporter assays, or RNA pull-down.

Below, we have summarized studies where lncRNA acts as a suppressor in its axes (Table 2).

The following study [108] focused on studying the pathways of cisplatin resistance regulation and revealed LINC00173 as an oncosuppressive lncRNA regulated by c-Myc, a well-known transcription factor (Table 2). In this study, a full axis was revealed to regulate the resistance of cancer cells to cisplatin treatment as well as other interesting patterns. All findings refer to lung adenocarcinoma (LUAD). Researchers first screened the Gene Expression Omnibus database and identified candidate LINC00173. Binding of c-Myc to the LINC00173 promoter, suppressing its expression, was first predicted using online PROMO algorithms and then confirmed by ChIP and a dual-luciferase test. LINC00173 lncRNA was shown to increase the BCL2 mRNA stability through the regulation of the miR-1275/PROCA1/ZFP36L2 axis, using several different methods such as transfection, in situ hybridization, qRT-PCR, Western blotting, and some others. The study demonstrated the downregulation of LINC00173 lncRNA in patients with LUAD resistant to cisplatin therapy associated with poor prognosis and conferred chemoresistance. The authors also revealed the suppression of c-Myc by LINC00173 lncRNA that led to miR-1275 overexpression. At the same time, miR-1275 correlated with PROCA1 expression that promoted apoptosis. Thus, this dynamic algorithm plays a pivotal role in LUAD development and cisplatin resistance.

Thus, suppressor lncRNAs and their interactomes inhibit proliferation, invasion, metastasis, activate apoptosis, and increase drug sensitivity.

All described lncRNAs require further investigations since suppressor lncRNAs may often act as oncogenes and vice versa. For instance, FOXD3-AS1 has a dual role and acts as an oncogene via the FOXD3-AS1/miR-127-3p/MDM2 axis, stimulating the progression of NSCLC and drug resistance [41] (Table 1), and via FOXD3-AS1/miR-150/SRCIN1, inhibiting the proliferation and invasion of H1299 cell lines [103] (Table 2).

All research groups used various available methods to validate the role of different identified lncRNA axes and clarify their role in NSCLC development. We noted, but did not fully describe, the functional diversity of various lncRNAs that can act as oncogenes, suppressors, or perform a dual role, such as FOXD3-AS1 [41,103]. We summarized the novel lncRNA-dependent ceRNA axes mediated by competitive endogenous RNAs with different functionalities in two tables.

### 2.2. LncRNAs Involved in Multiple ceRNA Axes

To illustrate the fact that a single lncRNA can be involved in many important interactions through the ceRNA mechanism, we chose several lncRNAs.

#### 2.2.1. Oncogenic lncRNA XIST

Quite a few studies identify XIST (X-inactive specific transcript) as one of the key “players” in NSCLC progression. This lncRNA acts as a functional inhibitor of X chromosome expression in mammals. At the same time, in various cancer types including NSCLC, XIST functions as an oncogene. Nevertheless, the mechanisms of XIST interactions and its role in various cellular processes in NSCLC are constantly being investigated. The study [102] focused on EMT as one of the key processes in malignant transformation of lung cells resulting from an impaired expression of involved genes. XIST was shown to be overexpressed in NSCLC and mediates an important pathway of EMT regulation in this cancer. Suppressing its expression downregulated ZEB2 through miR-367/miR-141. ZEB2 is responsible for triggering EMT via TGF-β activation. The inactivation of the ZEB2 suppressed TGF-β expression and consequently EMT responsible for progression and metastasis in NSCLC cells. Thus, the XIST/[miR-367/miR-141]/ZEB2 axis plays a key role in the progression and metastasis in NSCLC [102].

Another study focused on the relationship between XIST, miR-137, and the gene PXN (paxillin). PXN is known as an oncogene in NSCLC. This study confirmed PXN as a target for miR-137. In NSCLC cells, miR-137 was downregulated, whereas PXN was upregulated. The XIST overexpression resulted in the competitive binding to miR-137 reducing its expression. Thus, the authors established and confirmed the role of the XIST/miR-137/PXN axis in all processes involved in NSCLC development and progression: proliferation, maintenance of the cell cycle, migration, invasion, avoidance of apoptosis, and metastasis [100].

XIST was shown to be involved in signaling cascades during NSCLC. In cancer tissues and NSCLC cell lines, XIST overexpression enhanced the proliferation of tumor cells and prevented apoptosis, whereas XIST suppression resulted in the generation of reactive oxygen species (ROS) and the expression of the NLRP3 gene, which was involved in inflammasome formation and caspase 1 activation. This cascade activates pyroptosis in response to inflammatory signals. This process is controlled by the superoxide dismutase type 2 (SOD2), which also is a target of miR-335. Thus, the regulation of pyroptosis and proliferation is controlled by a complex XIST/miR-335/[SOD2/ROS] axis, a key stage in the development and progression of NSCLC [101].

Another study identified an axis significant for the resistance of tumor cells to platinum compounds. A comprehensive analysis, which included a bioinformatics search, database analysis, and experimental studies on cell lines, allowed for confirming the crucial role of the XIST/miR-520/BAX axis in regulating NSCLC resistance to cisplatin and promoting the ability of tumor cells to avoid apoptosis [99].

Autophagy is a conserved metabolic process crucial for cancer cells, as it ensures the ability of cancer cells to migrate and change the environment, invading the areas around. Autophagy in cancer cells is closely related to growth, chemoresistance, metastasis formation, and other processes. XIST was found to be significantly upregulated in various tumors compared to normal adjacent tissues [98]. A similar pattern was observed in the NSCLC cell lines. The downregulation of XIST led to significant diminution of the harmful effect of autophagy as well as the sequence of interactions mediating this process. As a result, the XIST/miR-17/ATG7 axis was demonstrated to be largely involved in regulating autophagy in NSCLC [98].

**Figure 1:**
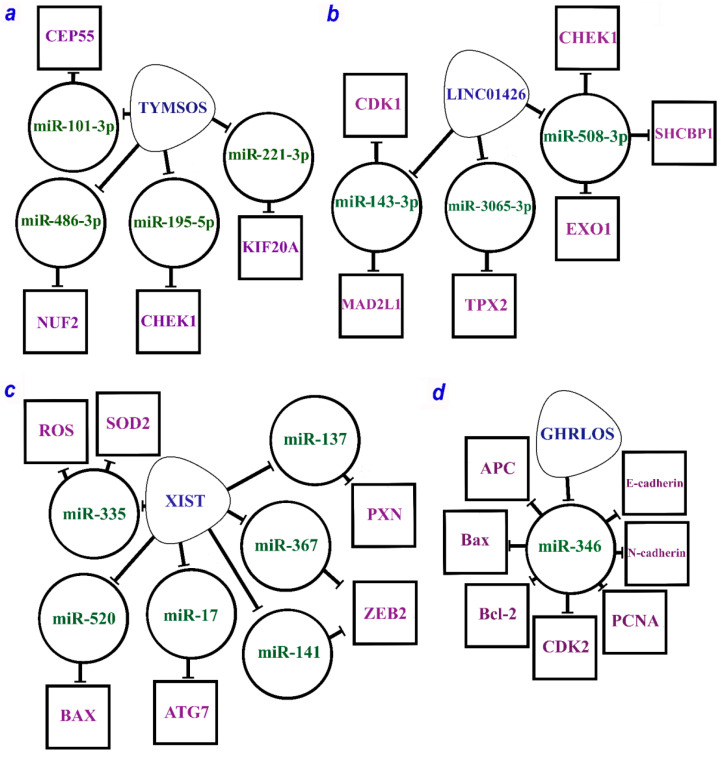
The multiple regulatory axes of lncRNAs are (**a**) TYMSOS (TYMS opposite strand RNA), (**b**) LINC01426 (long intergenic non-protein coding RNA 1426), (**c**) XIST (X-inactive specific transcript), and (**d**) GHRLOS (ghrelin opposite strand/antisense RNA) according to the studies [60,98,99,100,101,102,106]. lncRNAs are within soft triangles; miRNAs are within circles; proteins are within squares; blunt arrows indicate inhibitory interactions.

The combination of seven axes guided by the XIST lncRNA is given in Figure 1c.

#### 2.2.2. Oncogenic lncRNA MALAT1

When considering the well-studied MALAT1 (metastasis-associated lung adenocarcinoma transcript 1), it is necessary to focus on the latest results obtained for this lncRNA. This review discusses four studies that allowed us to chart its interactions. The earliest study was carried out on five cell lines and showed that in the presence of high levels of MALAT1, the expression of the interacting miRNA miR-124 was significantly reduced. At the same time, the direct target for miR-124 is mRNA STAT3, and its expression level is correlated with MALAT1 expression. Thus, the MALAT1/miR-124/STAT3 axis was identified to play a significant role in NSCLC progression [66].

Another study demonstrated the MALAT1/miR-145-5p/NEDD9 axis to be associated with the development of endothelial dysfunction in NSCLC as well as proliferation and further malignization of lung cells. The study was conducted in a comprehensive manner and included not only paired tissue samples but also cell lines and model animals, making the findings reliable [65].

The other two studies described the MALAT1/hsa-miR-515-5p/EEF2 and MALAT1/hsa-miR-515-3p/TRIM65 axes [63,64]. The miRNAs in these axes differ only in the end of the pre-miRNAs they originate from. Each axis was significantly involved in NSCLC development and associated with proliferation, migration, invasion, and many other systemic processes [63,64]. MALAT1 interactomes via miRNAs are shown below along with other mechanisms (see Section 3.2. below).

#### 2.2.3. Oncogenic lncRNAs TYMSOS and LINC01426

Furthermore, four axes were related to TYMSOS (the thymidylate synthetase opposite strand) lncRNA, which is potentially involved in the development and progression of NSCLC (Table 1, Figure 1a). Two axes, TYMSOS/hsa-miR-101-3p/CEP55 and TYMSOS/hsa-miR-195-5p/CHEK1, were validated as key pathways in the development and progression of NSCLC and its histological subtypes [60]. Several axes have been proposed for LINC01426 (long intergenic non-protein coding RNA 1426). In total, three miRNAs and six protein-coding genes regulated by LINC01426 involved in the development and progression of NSCLC have been identified so far (Figure 1b) [60].

All these axes described above are oncogenic, and a complete list of novel oncogenic axes involved in the development of NSCLC detected in the last 5 years is presented in Table 1.

#### 2.2.4. Suppressor of lncRNA GHRLOS

A complete list of the recently identified suppressor ceRNA axes is provided in Table 2. A study conducted on cell lines and NSCLC samples revealed several axes mediated by GHRLOS (ghrelin opposite strand/antisense RNA) lncRNA linking NSCLC development with proliferation, invasion, and apoptosis regulation. According to the earlier study [106], GHRLOS controls the interaction of miR-346 miRNA with major targets such as APC, Bax, Bcl-2, CDK2, E-cadherin, N-cadherin, and PCNA (Figure 1d). MiR-346 overexpression was observed in NSCLC, while the expression of GHRLOS and mRNAs of the abovementioned genes was significantly reduced. In the NSCLC cell lines, the miR-346 expression was inversely correlated with the expression of GHRLOS and a number of its mRNA targets, as it was expected. Thus, the GHRLOS/miR-346/[APC, Bax, Bcl-2, CDK2, E-cadherin, N-cadherin, PCNA] axes can be considered as critical regulators in the development of NSCLC [106].

Thus, over the last 3–5 years, many novel oncogenic and suppressor lncRNAs were identified in NSCLC acting in accordance with the ceRNA model (Table 1 and Table 2). It includes five lncRNAs with multiple axes, indicating the multifunctional role of lncRNAs and their potential ability to dynamically switch to the interactions necessary for a normal or cancer cell.

## 3. Alternative Mechanisms of lncRNAs in Regulation of Target Genes in NSCLC

Although there are many well-established examples of lncRNAs involved in the regulation of protein-coding gene expression by the ceRNA model, several other alternative mechanisms of lncRNA action in NSCLC were identified as well. These mechanisms are mediated by both direct action of lncRNA on target mRNA and the function of RNA-binding proteins or heterogeneous nuclear ribonucleoproteins.

### 3.1. Effects of lncRNAs on Target mRNAs via Direct Binding

Data on the direct effect of lncRNA on target mRNA are summarized in Table 3.

#### 3.1.1. Downregulation of Target mRNAs

LncRNAs can either increase or decrease the level of mRNAs and proteins as translation products (Table 3). TUSC8 (tumor suppressor candidate 8) is an example of downregulated in NSCLC lncRNA with tumor suppressive function. TUSC8 overexpression abated NSCLC cell proliferation by inhibiting the vascular endothelial growth factor A (VEGFA) (Table 3). Direct binding of TUSC8 to the 3’UTR of VEGFA mRNA, identified by bioinformatics analysis, was confirmed by double luciferase reporter test [116]. Moreover, TUSC8 upregulation increased the sensitivity of NSCLC cells to cisplatin, at least partially, through the inhibition of VEGFA.

HOXA-AS3 (HOXA cluster antisense RNA 3) is another example of an oncogenic lncRNA that directly inhibits target mRNA. HOXA-AS3 downregulates the oncosuppressor gene HOXA3 (homeobox A3) (Table 3). Moreover, direct binding of HOXA-AS3 to both HOXA3 mRNA and protein was demonstrated using RNA pull-down assay, mass spectrometry, and RNA immunoprecipitation. HOXA-AS3 upregulation promotes EMT via HOXA3 downregulation and increases cisplatin resistance, as demonstrated both in vitro and in vivo [117].

**Table 3:** Direct action of lncRNAs on mRNAs in NSCLC progression.

Mechanisms, Axes	LncRNAs/Axes in Processes, Pathways, Prognosis, Survival, and Drug Resistance	Ref.
**LncRNA/mRNA, protein**		
**HIF2PUT**↓ov-ex/HIF-2a↓mRNA, protein	Inhibits NSCLC proliferation, invasion	[118]
**HOTAIR**↑/CCL22↓mRNA, protein →CCL22-sign↓→Treg↓	Promotes invasion, immune evasion; poor prognosis	[119]
**HOXA-AS3**↑/HOXA3↓mRNA, protein	Enhances EMT, drug resistance in vitro/in vivo	[117]
**NBR2**↓ov-ex/Notch1↓mRNA, protein	Inhibits EMT, progression, Notch1 sign	[120]
**lncRNA-NEF**↓ov-ex/GLUT1↓mRNA, protein	Inhibits cell proliferation, glucose uptake	[121]
**STXBP5-AS1**↓ov-ex/STXBP5↓mRNA, protein; **STXBP5-AS1**↓ov-ex/AKT1↓mRNA, protein	Inhibits cell proliferation, migration, invasion, and PI3K/AKT p-w	[122]
**TUSC8**↓ov-ex/VEGFA↓(3′UTR) mRNA	Inhibits NSCLC; better OS, cisplatin sensitivity	[116]
**WT1-AS**↓ov-ex/TGF-β1↓mRNA, protein	Inhibits cancer cell stemness; improves survival	[123]
**LncRNA→mRNA, proteins**		
**AWPPH**↑→TGF-β1↑mRNA (in blood)	Promotes cell migration, invasion, and distant recurrence	[124]
**BLACAT1**↑→Cyclin D1↑protein	Enhances cisplatin resistance	[125]
**CASC2**↑→PERK↑mRNAstab, protein /eIF2α↓protein(phosph)→CHOP↑protein	Inhibition of NSCLC, promotion of radiosensitivity, and endoplasmic reticulum stress p-w in irradiated NSCLC cells	[126]
**DSCAM-AS1**↑→BCL11A↑mRNA, protein	Promotes cell migration, invasion, and poor OS	[127]
**FEZF-AS1**↑→FEZF1↑mRNA	Correlation with advanced stages	[128]
**HOXA-AS2**↑→IGF2↑mRNA, protein	Promotes cell migration, invasion, and metastasis	[129]
**HOXC-AS2**↑↔HOXC13↑mRNA	Enhances proliferation, migration, and EMT	[130]
**LALTOP**↑→Top2α↑mRNAstab	Enhances NSCLC progression, cell migration	[131]
**LINC01288**↑→IL-6↑mRNAstab→pSTAT3↑protein	Promotes migration, metastasis in vitro/ in vivo, STAT3 sign	[132]
**MALAT1**↑→SOX9↑mRNA, protein	Enhances chemoresistance; poor OS	[133]
**NORAD**↑→CXCR4↑CXCL12↑protein→ RHOA,ROCK1,ROCK2,LIMK1,LIMK2,P-CFL↑	Activates proliferation, migration, invasion, RhoA/ROCK sign, in vitro/in vivo	[134]
**SENCR**↑→FLI1↑mRNA, protein	Promotes tumor growth, cisplatin resistance	[135]
**SFTA1P**↑↔TAZ↑mRNA,protein↔YAP-TAZ-TEAD↑	Promotes proliferation in vitro/in vivo; Hippo-YAP/TAZ sign p-w	[136]
**SNHG7**↑→MRD1, BCRP↑mRNA,protein; **SNHG7**↑→P-gp, PI3K,AKT,mTOR↑protein	Induces cisplatin resistance, PI3K/AKT/mTOR sign p-w	[137]
**TMPO-AS1**↑→TMPO↑mRNAstab	Promotes NSCLC progression in vitro/in vivo	[138]
IL-6↑mRNA→**ZEB2-AS1**↑→pSTAT1↑protein	Promotes migration, metastasis, and poor OS	[139]
**ZNF205-AS1**(pr)↑↔EGR4↑mRNA, stab	Promotes tumor cell growth; poor prognosis	[140]

Note: OS—overall survival; ov-ex—overexpression; phosph—phosphorylation; pr—promoter; p-w—pathway; stab—stability; sign—signaling; ↑ ↓—increase or decrease in expression or stabilization; →—activation; /—inhibition; ↔—a positive feedback.

Note that in Table 3 we present the works that employed complex methods including bioinformatics and traditional experimental approaches based on NSCLC cell lines (in vitro), clinical samples, or animal models (in vivo). We also included works utilizing modern methods to demonstrate lncRNA binding to its targets, such as RNA pull-down analysis, RNA immunoprecipitation (RIP), or a double luciferase reporter assay.

#### 3.1.2. Upregulation of Target mRNAs

Both TMPO-AS1 (TMPO antisense transcript 1) lncRNA and TMPO (thymopoietin) mRNA were overexpressed in NSCLC cell lines and tissues and promoted cell proliferation, colony formation, migration, and invasion of NSCLC cells, demonstrating the oncogenic function of TMPO-AS1 and TMPO [138]. TMPO-AS1 and TMPO exon 1 were shown to have an area of overlap. Overexpression of the cloned overlap region increased the mRNA and protein levels of TMPO. In addition, TMPO-AS1 increased the stability of TMPO mRNA through antisense pairing [138]. Thus, this oncogenic lncRNA elevated the level of the target mRNA as well as its stability (Table 3).

LALTOP (lung-cancer-associated lncRNA targeting topoisomerase II alpha, Top2α) is a novel lncRNA upregulated in NSCLC tissues and cell lines. The authors identified the Top2a transcript as the interacting partner of LALTOP through predicted binding sequences and confirmed the interaction using RNA–RNA in vitro binding assays and fluorescence in situ hybridization (FISH) assays [131]. Moreover, LALTOP overexpression increased the stability of Top2a mRNA. A positive correlation between LALTOP and Top2α mRNA expressions in clinical samples was demonstrated. LALTOP significantly promoted the proliferation and migration of A549 and H1793 NSCLC cells, while antisense oligonucleotides which target LALTOP inhibited the malignant phenotypes of NSCLC. Thus, LALTOP is an oncogenic lncRNA that promotes the progression of NSCLC by increasing the level and stability of Top2a mRNA [131].

ZNF205-AS1 transcript (ZNF205 (zinc finger protein 205) antisense RNA 1) was shown to exert a dual effect on the target gene mRNA, namely increasing both the level and stability of early growth response 4 (EGR4) mRNA [140] (Table 3). Direct RNA–RNA binding between ZNF205-AS1 and EGR4 mRNA was confirmed by the luciferase reporter assay. Moreover, the predicted binding of EGR4 mRNA to the ZNF205-AS1 promoter region was determined by chromatin immunoprecipitation (ChIP) and dual luciferase reporter assays. EGR4 also activated ZNF205-AS1 transcription through interaction with the ZNF205-AS1 promoter region, establishing a positive feedback loop between ZNF205-AS1 and EGR4. Oncogenic ZNF205-AS1 and EGR4 promoted NSCLC cell and tissue growth, as shown by gain-of-function and loss-of-function assays in both in vitro and in vivo experiments. Thus, a positive feedback loop between ZNF205-AS1 and EGR4 mRNA ensured the oncogenic functions of these genes in NSCLC [140].

A positive feedback loop was also shown for lncRNA SFTA1P (surfactant-associated 1 pseudogene) and mRNA of TAZ (encoding the protein tafazzin). Moreover, SFTA1P binding to TAZ mRNA was demonstrated to increase TAZ protein translation but not the level of TAZ mRNA or its stability [136]. SFTA1P enhanced the proliferation and tumorigenic potential of NSCLC cells, while the loss of SFTA1P inhibited tumor growth both in vitro and in vivo. The sequence adjacent to the SFTA1P transcription start site (TSS) contains seven consensus TEAD binding sites, and SFTA1P can exhibit YAP/TAZ/TEAD-dependent promoter activity in NSCLC cell lines. The simultaneous knockdown of both YAP and TAZ led to a significant decrease in SFTA1P expression in the H1299 NSCLC cell line. Thus, SFTA1P was shown to be a novel transcriptional target of YAP/TAZ/TEAD. A comprehensive study using various methods, including loss- and gain-of-function approaches, luciferase tests, and gene knockout, allowed identifying a positive feedback loop between SFTA1P and the Hippo-YAP/TAZ signaling pathway [136].

Other examples of longer axes mediated by predominant activation of a target mRNA or protein by oncogenic lncRNAs include two mechanisms that upregulate STAT1 and STAT3 (signal transducer and transcription activator 1 and 3) via interleukin-6 (IL-6) and LINC01288 and ZEB2-AS1 lncRNAs (Table 3). As shown in [132], the long intergenic non-coding RNA LINC001288 is overexpressed in NSCLC tissues and cells and stimulates cell migration, tumor growth, and metastasis both in vitro and in vivo. According to gene set enrichment analysis (GSEA) data, LINS001288 exerted the greatest effect on the JAK-STAT signaling pathway. Direct binding of LINC01288 to IL-6 mRNA with upregulation of its level was predicted by transcriptome sequence analysis and confirmed by RT-qPCR, RIP, and RNA pull-down assays. Moreover, a strong correlation between IL-6 mRNA and LINC01288 was observed in NSCLC tissues. LINC01288 inhibited the degradation of the IL-6 transcript by α-amanitin, thereby increasing the stability of IL-6 mRNA. Furthermore, LINC01288 increased the phosphorylated levels of STAT3 protein via autocrine induction of IL-6 [132]. This evidence indicates the existence of a novel signaling axis in NSCLC (Table 3) implicating a complex crosstalk between lncRNA and cancer progression.

The oncogenic antisense lncRNA ZEB2-AS1 (zinc finger E-box binding homeobox 2 antisense RNA 1) is known to stimulate proliferation and inhibit apoptosis in NSCLC. ZEB2-AS1 overexpression is accompanied by the IL-6 induction [139]. In A549 cells, phosphorylated pSTAT1 was upregulated by IL-6, which stimulated NSCLC progression by activating pSTAT1. The pSTAT1/3 levels in IL-6-treated A549 cells were detected by Western blotting; the interaction between ZEB2-AS1 and IL-6 mRNA was evaluated by RT-qPCR; and the interaction between ZEB2-AS1 and pSTAT1/3 was validated by ChIP assay [139]. The IL-6 mRNA↑→ZEB2-AS1↑→pSTAT1/3↑ axis contributes to NSCLC metastasis and correlates with poor overall survival (Table 3). Thus, there is evidence that NSCLC progression is promoted by both LINC01288 and ZEB2-AS1 and that the underlying mechanisms involve IL-6 and STAT1/3 family proteins.

Advanced inoperable NSCLC is most commonly treated using radiation therapy. Radiotherapy induces endoplasmic reticulum (ER) stress and ultimately activates apoptosis. A tumor suppressor lncRNA CASC2 (cancer susceptibility candidate 2) was reported to enhance irradiation-induced ER stress in NSCLC cells via protein kinase-like ER kinase (PERK) signaling [126]. In irradiated NSCLC cells, CASC2 overexpression stimulated cell apoptosis through PERK upregulation, mainly by increasing PERK mRNA stability. Of note, CASC2 increased PERK protein levels in irradiated NSCLC cell lines but did not affect them in the absence of irradiation. The ER transmembrane protein kinase PERK suppresses protein synthesis via inhibiting eIF2α (eukaryotic translation initiation factor 2-alpha) by serine 51 phosphorylation. In turn, eIF2α inhibition elevates the expression of C/EBP (CCAAT/enhancer-binding protein) homologous protein (CHOP), a DNA-binding transcription factor involved in ER stress and the induction of DNA damage and apoptosis. Various approaches such as lentiviral vector transfection, Western blotting, and double luciferase testing [126] allowed for identifying the CASC2 axis, the mechanism underlying the CASC2-dependent stimulation of PERK signaling and NSCLC radiosensitivity (Table 3).

### 3.2. Action of lncRNAs Mediated by RNA-Binding Proteins (RBPs)

LncRNAs can alter gene expression and/or its functions by acting as miRNA spongers, via a direct interaction of lncRNAs with mRNAs or binding to RNA-binding proteins (RBPs) [141,142]. RBPs were shown to regulate mRNA expression and stability at the post-transcriptional level [19]. RBPs combine a flexible structure with a versatile RNA-binding domain [19,21]. These properties allow RBPs to engage in highly dynamic interactions both with other proteins as well as with coding and non-coding RNAs, leading to ribonucleoprotein complexes (RNPs) being formed. RNPs regulate RNA splicing, polyadenylation, stability, localization, translation, and degradation [20,21].

In NSCLC, lncRNAs can regulate the levels and stability of target mRNAs by binding RBPs to form RNP complexes, as was demonstrated in a number of examples. We identified 17 lncRNAs that function according to this mechanism. Six of them function through IGF2BP1/2/3, the effects of three lncRNAs are mediated by HuR/ELAVL1, and the action of three lncRNAs depends on heterogeneous nuclear ribonucleoproteins such as RBPs (hnRNPD (AUF1), hnRNPK, hnRNPU), and the function of five other lncRNAs requires five different RBPs: EIF4A3, FBL, UPF1, WDR5, and YTHDF1 (Table 4).

All data on the interaction between RNA-binding proteins with lncRNAs and mRNAs, presented in Table 4, were validated using the methods that confirm direct binding, such as RIP, ChIP, RNA pull-down, luciferase tests, etc.

#### 3.2.1. IGF2BP1/2/3 as RBP Mediator

IGF2BP1/2/3 (insulin-like growth factor 2 mRNA-binding protein 1/2/3) proteins are the most common RBPs mediating lncRNA regulatory functions. RBPs promote cancer cell proliferation, migration, and invasion, and their oncogenic functions are mediated by post-transcriptional regulation of mRNA stability and translation [21]. Here, we describe the IGF2BP1/2/3-dependent regulatory functions of six lncRNAs in NSCLC.

LINC01232 (long intergenic non-protein coding RNA 1232) is highly expressed in NSCLC cells and promotes cell stemness. This was suggested by estimating the mRNA and protein levels of stem cell markers (OCT4, Nanog, CD133, SOX2, and SOX4) and the sphere formation assay [145]. Two binding sites for the transcription factor FOXP3 (forkhead box P3) in the LINC01232 promoter region were predicted using DNA motifs from the JASPAR database. Luciferase reporter and ChIP assays were employed to confirm the direct binding between FOXP3 and the LINC01232 promoter, which activates LINC01232 transcription. LINC01232 was shown to activate the TGF-β signaling cascade by assessing the levels of the major proteins involved in this pathway (TGF-β1, α-SMA, SMAD2, and SMAD3) by Western blotting. StarBase (http://starbase.sysu.edu.cn/index.php, accessed on 19 June 2023) and the GEPIA database identified RBPs for LINC01232 and transforming growth factor beta receptor 1 (TGFBR1) as the critical modulators of the TGF-β signaling pathway, which could be activated by LINC01232. Three candidate RBPs (IGF2BP2, IGF2BP3, and DKC1) activated in NSCLC tissues and potentially associated with TGFBR1 and LINC01232 were selected for further analysis. Using RIP and RNA pull-down assays, IGF2BP2 was identified as the only RBP that interacted with both TGFBR1 and LINC01232. IGF2BP2 and LINC01232 were co-localized in the cytoplasm, as shown by FISH and FISH-IF assays. RT-qPCR and Western blotting revealed a positive association between LINC01232 and TGFBR1, indicating that IGF2BP2 overexpression can upregulate TGFBR1 expression, increasing both TGFBR1 mRNA and protein levels. In summary, FOXP3-activated LINC01232 in the RNP complex with IGF2BP2 was shown to increase the stability of TGFBR1 mRNA and therefore stimulate the TGF-β signaling pathway, facilitate the stemness of NSCLC cells, and induce the M2 polarization of macrophages [145].

The lncRNA MNX1-AS1, also known as LOC645249 and CCAT5 (colon-cancer-associated transcript 5), is an antisense transcript of the motor neuron and pancreas homeobox protein 1 MNX1. MNX1-AS1 is highly expressed in NSCLC and exhibits oncogenic properties [147]. Its transcriptional activation involves transcription factor c-Myc and the copy number amplification mechanism. MNX1-AS1 overexpression is associated with poor clinical outcomes in NSCLC patients. It is shown to promote cell proliferation and colony formation in vitro and tumor growth in vivo. In NSCLC, MNX1-AS1 binds to IGF2BP1 and causes phase separation of IGF2BP1, which facilitates the IGF2BP1 binding to the 3’UTR of c-Myc and E2F1 mRNAs and enhances the stability of these mRNAs [147]. These interactions form a positive feedback loop that drives IGF2BP1 phase separation and promotes c-Myc and E2F1 signaling, cell cycle progression, and proliferation of NSCLC cells (Table 4).

Ectopic overexpression of lnc-THOR in NSCLC elevated the expression of IGF2BP1 target mRNAs (IGF2, Gli1, Myc, and SOX9), enhancing its mRNA stability (Table 4). The direct binding of lnc-THOR to the IGF2BP1 protein was validated using RIP and RNA pull-down assays [148]. Through this axis, lnc-THOR stimulates the proliferation, migration, and invasion of NSCLC cells in vitro and tumor growth in vivo [148].

There is evidence for the specific contribution of linc-SPRY3 (sprouty protein homolog 3)-2/3/4, the Y chromosome transcript, to the radiation response of male NSCLC [144]. Predicting RBP motifs using a bioinformatic approach allowed for identifying IGF2BP3 as a binding partner for linc-SPRY3-2/3/4. Direct interaction of IGF2BP3 with linc-SPRY3-2/3/4 was validated using UV cross-linking and immunoprecipitation (CLIP) assays. Linc-SPRY3-2/3/4 reduced the stability of the mRNA of anti-apoptotic and oncogenic HMGA2 and c-MYC targets by binding IGF2BP3 and increased the radiosensitivity of male NSCLC [144]. Thus, suppressor lncRNAs linc-SPRY3-2/3/4 mediated with IGF2BP3 increased the response to radiotherapy on the Y chromosome (Table 4).

RBPs can also act as a N(6)-methyladenosine (m^6^A) reader or m^6^A writer proteins in mRNA methylation via (m^6^A)-methyltransferase complex [160,161]. LncRNA LCAT1 (lung-cancer-associated transcript 1) was shown to bind and stabilize the m^6^A-IGF2BP2 reader protein, preventing its lysosomal degradation [143]. Stabilized m^6^A-IGF2BP2 then facilitated the translation and stability of cell division cycle 6 protein (CDC6) mRNA, which promoted the proliferation, survival, and migration of NSCLC cells. RNA pull-down, mass spectrometry, and RIP were used to validate the direct interactions.

Using the CRISPR/Cas9 system, METTL3, the core m^6^A methyltransferase, was found to be involved in interactions between IGF2BP2 protein and CDC6 mRNA dependent on m^6^A modifications. Therefore, METTL3 (m^6^A-writer) and m^6^A-IGF2BP2 (m^6^A-reader) promoted the upregulation and stabilization of CDC6 by LCAT1, which enhanced the progression of NSCLC and impaired patient survival [159] (Table 4).

Another study showed an activating and stabilizing effect of IGF2BP2 as both RBP and m^6^A-reader in the axis with MALAT1 lncRNA and ATG12 (autophagy-related 12) protein [146] (Table 4). First, IGF2BP2 mRNA level was elevated in primary NSCLC tissues and positively correlated with poor overall survival (OS) and disease-free survival (DFS). Its ectopic expression and knockdown in NSCLC cell lines and in vivo showed that IGF2BP2 promoted NSCLC cell proliferation and tumor growth. We would like to emphasize that the activating and stabilizing effect of IGF2BP2 is exerted directly on the lncRNA MALAT1 as well as indirectly via the formation of m^6^A-MALAT1 by m^6^A-RNA methylation. Direct binding between IGF2BP2 and MALAT1 was validated using the RIP-PCR assay, while mRIP-PCR with m^6^A antibody confirmed the m^6^A modification of MALAT1. Western blotting showed that the overexpression of IGF2BP2 binding MALAT1 increased the level of ATG12 protein, a key downstream target of MALAT1 [146]. Thus, IGF2BP2 was demonstrated to enhance MALAT1 stability through m^6^A modification promoting the protein expression of its downstream target ATG12, thereby facilitating NSCLC progression and reducing patient survival (Table 4).

#### 3.2.2. HuR/ELAVL1 as RBP-Mediator

Post-transcriptional gene regulation relies on hundreds of RNA-binding proteins. One of the mechanisms of post-transcriptional gene regulation in mammalian cells is the rapid degradation of mRNAs containing AU-rich elements (ARE) in their 3’-UTR [162]. ELAVL1 (Drosophila ELAV-like RNA-binding protein 1) or HuR (human antigen R) belongs to the ELAVL family of RBPs that contains several RNA-binding domains. Overexpressed HuR/ELAVL1 selectively binds to cis-acting ARE and stabilizes ARE-containing mRNAs in cells. Human RBP HuR/ELAVL1 is a conserved mRNA stability regulator [162,163].

HuR/ELAVL1 was reported as the common RBP of lncRNA MCF2L-AS1 (MCF2 cell-line-derived transforming sequence-like 2 antisense RNA 1) and cyclin D1 (CCND1) [150]. The interactions of MCF2L-AS1 and CCND1 mediated by HuR/ELAVL1 revealed a new potential mechanism for NSCLC progression and development of resistance to the widely used drug gefitinib. Cyclin D1 (CCND1) is an oncoprotein involved in the regulation of the cell cycle and the transition from the G1 phase to the S phase. Both the lncRNA MCF2L-AS1 and CCND1 were overexpressed in NSCLC cell lines. In addition, MCF2L-AS1 was upregulated by the transcription factor E2F1, which binds to the MCF2L-AS1 promoter region, as predicted by JASPAR data [150]. The direct interaction between them was confirmed by luciferase and ChIP assays [150], while the direct interaction between MCF2L-AS1, CCND1, and HuR/ELAVL1 was validated using RNA pull-down and RIP assays [150]. These experiments showed that MCF2L-AS1, activated by the binding of transcription factor E2F1 to the promoter region in complex with HuR/ELAVL1, interacts with the CCND1 mRNA, increasing its stability, driving NSCLC progression, and inducing patient resistance to gefitinib (Table 4).

An intriguing mechanism reducing NSCLC stemness was found for the suppressor lncRNA FENDRR (FOXF1 adjacent non-coding developmental regulatory RNA), also known as FOXF1-AS1. It is mediated by the inhibition of oncogenic multidrug resistance gene 1 (MDR1) mRNA and RBP HuR [149]. FENDRR lncRNA expression is decreased in NSCLC tissues and cells, and its overexpression abates the stemness of NSCLC cells. The latter was confirmed by stemness marker (CD34 and CD133) expression analysis and the capacity of cells for spheroid formation. Direct binding of lncRNA FENDRR to MDR1 3’UTR was shown using such methods as the luciferase reporter and RIP assays [149]. MDR1 mRNA stability was measured in NSCLC cells in the presence of actinomycin D with or without FENDRR overexpression. MDR1 mRNA stability was significantly decreased upon FENDRR overexpression. Thus, lncRNA FENDRR directly binds to the MDR1 3′UTR and reduces MDR1 mRNA stability [149].

RBP HuR can promote the stabilization of target transcripts and bind to mRNA 3′UTR with AU-rich elements. Direct interaction of HuR with the MDR1 3’UTR was validated using luciferase reporter and RIP assays [149]. In addition, RBP HuR increased the expression and stability of MDR1 mRNA. Moreover, lncRNA FENDRR was shown to compete with HuR for binding to MDR1 3′-UTR, and its overexpression could partially prevent HuR binding to MDR1 3’UTR. Thus, lncRNA FENDRR and RBP HuR, two crucial epigenetic regulators capable of binding to 3’UTR of MDR1, were demonstrated to exert opposite effects and compete with each other [149]. FENDRR lncRNA suppressed NSCLC cell stemness by inhibiting MDR1, whereas RBP HuR, which competed with FENDRR, played the opposite role (Table 4).

HuR RBP is involved in elevating the level and stability of PD-L1 (programmed cell death 1 ligand 1) and USP8 (ubiquitin-specific processing protease 8) mRNA by lncRNA SNHG12 (small nucleolar RNA host gene 12). As PD-L1 stabilization contributes to the evasion of the immune response in NSCLC, targeting this pathway can be promising for NSCLC immunotherapy [151]. The recently discovered immune checkpoint (PD-L1/PD-1) blocks production of antibodies and cytokines in cancer, thus impairing immune cell activation and reducing the immune response towards tumor cells. HuR at the post-transcriptional level increased the level and stability of USP8 mRNA and facilitated translation of USP8 protein, promoting the proliferation, migration, and invasion of NSCLC cells. USP8 is a deubiquitinase that can inhibit ubiquitin-dependent degradation and increase the stability of oncoproteins. SNHG12 is highly expressed in NSCLC tissues and cells [151]. An increase in the SNHG12 lncRNA expression was shown to be associated with a shorter overall survival of patients with NSCLC (Kaplan–Meier curves). The half-life of USP8 and PD-L1 mRNAs in NSCLC cells was assessed by RT-qPCR. ChIP assay confirmed the interaction between USP8 and PD-L1 proteins. USP8 was shown to stabilize PD-L1 through deubiquitination. Direct binding of SNHG12 to HuR, as well as binding of PD-L1 and USP8 to HuR, was predicted using RNA–protein interaction prediction (RPISeq) (http://pridb.gdcb.iastate.edu/RPISeq/, accessed on 19 June 2023) and validated by RIP assay [151]. SNHG12 was shown to elevate the expression and stability of PD-L1 and USP8 mRNA as well as the level of translation of the USP8 protein due to its binding to HuR. Deubiquitination of PD-L1 suppresses immune CD8+ T cells and contributes to the escape of NSCLC from the immune response (Table 4).

#### 3.2.3. Heterogeneous Nuclear Ribonucleoproteins with RBP Function

Heterogeneous nuclear ribonucleoproteins (hnRNPs) are involved in many processes, including alternative splicing, transcription and translation regulation, and mRNA stabilization [164]. Possessing RBP properties, hnRNPs can mediate the regulatory effect of lncRNA on target mRNA or play a supportive role. In NSCLC, lncRNAs function together with three heterogeneous nuclear ribonucleoproteins: hnRNPD (or AUF1), hnRNPK, and hnRNPU.

AUF1 (AU-rich element RNA binding/degradation factor 1) is a heterogeneous nuclear ribonucleoprotein (hnRNPD) recognized as a classical RBP. AUF1 has a positive effect on the immune response towards NSCLC and demonstrates tumor suppressor properties. AUF1 functions as an antagonist of the oncogenic lncRNA SChLAP1 preventing SChLAP1-dependent stabilization of PDL-1 mRNA which allows NSCLC cells to evade the immune response [152]. LncRNA SChLAP1 (SWI/SNF complex antagonist associated with prostate cancer 1) is upregulated in NSCLC and promotes NSCLC cell proliferation, migration, and invasion [152], while its knockdown represses tumor growth and metastasis in vivo. SChLAP1 also facilitates immune evasion by enhancing PD-L1 mRNA stability and might inhibit AUF1 as a negative regulator of mRNA stability. SChLAP1 binding to AUF1 was predicted from the RNA-Protein Interaction Prediction (RPISeq) database (http://pridb.gdcb.iastate.edu/RPISeq/, accessed on 19 June 2023) and validated using the RIP and RNA pull-down assays [152]. SChLAP1 binding to AUF1 was shown to decrease AUF1 level and prevent AUF1 from suppressing PD-L1 mRNA stability. SChLAP1 overexpression decreased AUF1 enrichment in the 3’UTR region of PD-L1. Of note, PD-L1 upregulation induced by SChLAP1 overexpression was abolished upon AUF1 overexpression [152]. As a result, SChLAP1 overexpression elevated the levels of PD-L1 mRNA and protein. Thus, the oncogenic lncRNA SChLAP1 and RBP AUF1 compete for effects on the expression and stability of PD-L1 at the post-transcriptional level. PD-L1 is usually highly expressed on the surface of tumor cells repressing CD8+ T cell function. In summary, SChLAP1 binds to AUF1, reducing the interaction between AUF1 and the PD-L1 3’UTR and thus increasing PD-L1 mRNA stability and expression, which in turn represses CD8+ T cell function and facilitates tumor cell immune escape (Table 4).

Heterogeneous nuclear ribonucleoprotein hnRNPK is a key RNA-binding protein with oncogenic functions. In NSCLC, it stimulates the transcription of its target, the c-MYC oncogene, by participating in the binding of RNA polymerase II (RNA Pol II) to c-MYC [153]. The lncRNA DIO3OS (DIO3 opposite strand upstream RNA, or antisense lncRNA transcribed from the DIO3 (type 3 iodothyronine deiodinase) imprinted locus) in NSCLC directly interacts with hnRNPK, repressing hnRNPK binding to MYC DNA and mRNA, and inhibits MYC transcription and translation [153]. Furthermore, the lncRNA DIO3OS can suppress CDC25A, a downstream MYC target (Table 4). Ectopic expression of DIO3OS lncRNA suppresses NSCLC tumorigenesis and metastasis in vitro and in vivo. However, all these effects can be abolished by methylation of the CpG-456 dinucleotide gene encoding DIO3OS lncRNA, which can occur in NSCLC. The direct interactions between DIO3OS lncRNA, hnRNPK, c-MYC, CDC25A, and RNA Pol II in NSCLC were confirmed by complex methods such as dual-luciferase reporter assay, RNA pull-down, RIP and ChIP assays, and Western blotting [153].

RBP hnRNPU, which exhibits a suppressor function, mediates the interactions of suppressor LIMD1 (LIM domains-containing 1) mRNA with suppressor lncRNA LIMD1-AS1 (LIMD1 antisense RNA 1), suppressing NSCLC growth in vitro and in vivo [154] (Table 4). Moreover, LIMD1-AS1 interaction with hnRNPU stabilized LIMD1 mRNA. Unlike hnRNPK [153] and hnRNPD AUF1 [152], which prevented lncRNA from interacting with mRNA via competitive binding, hnRNPU promoted the interaction of lncRNA LIMD1-AS1 with the mRNA target LIMD1. Their direct binding was confirmed using RIP and pull-down assays in [154].

#### 3.2.4. Other RBPs (FBL, EIF4A3, UPF1, WDR5, YTHDF1/2/3) as Mediators of lncRNAs

FBL, EIF4A3, UPF1, WDR5, and YTHDF1/2/3 also act as RBP mediators of lncRNA action on protein-coding genes in NSCLC.

FBL was found to be an RBP mediator in the interaction between FAM83A antisense transcript 1 (FAM83A-AS1) and FAM83A pre-mRNA (family with sequence similarity 83 member A). Both FAM83A and FAM83A-AS1 are typical pro-tumor genes and are overexpressed in NSCLC, they activate cell migration and metastasis in vitro, and their levels correlate with OS and PFS [155]. RNase protection assay and RT-qPCR were used to detect duplex formation within the FAM83A pre-mRNA and FAM83A-AS1 overlapping region identified between exon 3 and exon 4 of the FAM83A gene. FAM83A-AS1 was shown to enhance FAM83A stability due to formation of the RNA–RNA duplex. The RNA pull-down and RIP assays and RT-qPCR showed the interaction of FBL RBP both with FAM83A and FAM83A-AS1 [155]. This indicates that an increase in FAM83A mRNA stability occurs not only due to the RNA–RNA interaction but also due to a triple RNP complex formed with the FBL protein [155]. Moreover, FAM83A-AS1 promotes NSCLC progression via ERK signaling pathways and metastasis by increasing FAM83A expression through FAM83A-AS1 to FAM83A pre-mRNA binding by forming an RNA/mRNA heteroduplex. RBP FBL binds the duplex enhancing FAM83A mRNA stability (Table 4).

Various antisense genes are involved in regulating their neighboring genes. Another example of RBP-dependent regulation of a protein-coding gene by an adjacent complementary antisense transcript is the TM4SF19 gene (transmembrane 4 L six family member 19). It is regulated by the conservative lncRNA TM4SF19-AS1 encoded by the TM4SF19 gene cluster, located at chromosome 3q29, and depends on WDR5 (WD repeat-containing protein 5) [158]. TM4SF19-AS1, like TM4SF19, was both upregulated in cells and patients’ tissues of lung squamous cell carcinoma (LSCC). Like TM4SF19, TM4SF19-AS1 can promote LSCC cell proliferation and adhesion. Moreover, there was a markedly positive correlation between TM4SF19-AS1 and TM4SF19 levels [158].

Furthermore, TM4SF19-AS1 activated TM4SF19 via WDR5 binding and inducing demethylation of the TM4SF19 promoter region. WDR5 was shown to exert two functions. WDR5 is a key subunit of the chromatin remodeling complex MLL1 (mixed-lineage leukemia 1), capable of enforcing active chromatin. It is also an RBP subunit, as it contains an RNA-binding pocket which allows lncRNA binding and regulates their target genes [165]. Moreover, WDR5 can be introduced into the promoter region of its target gene by lncRNAs to demethylate the target gene promoter and activate target gene expression. Similar results were obtained for lncRNA TM4SF19-AS1, which interacted with WDR5 and delivered it to the TM4SF19 promoter causing subsequent promoter demethylation and TM4SF19 upregulation [158].

This mechanism was validated by several different approaches. First, FISH showed that lncRNA TM4SF19-AS1 is localized in the nucleus and can interact with DNA. The direct interaction of WDR5 with the TM4SF19 gene promoter was validated using ChIP with TM4SF19-specific antibodies. In addition, the methylation status of the TM4SF19 promoter region was determined using a methylation-specific PCR (MSP) assay. TM4SF19 promoter methylation was reduced in LSCC cell lines, but its methylation level was elevated after si-TM4SF19-AS1 treatment. WDR5 binding to TM4SF19-AS1 was validated by RIP with WDR5-specific antibodies [158].

In summary, there is substantial evidence that in LSCC, the interaction between TM4SF19-AS1 and WDR5 reduced the TM4SF19 methylation leading to TM4SF19 upregulation (Table 4). These data represent a rare example of the interaction of lncRNAs with DNA, i.e., with the promoter of a protein-coding gene mediated by RBP.

Eukaryotic translation initiation factor 4A3 (EIF4A3) is a well-characterized RBP that regulates the expression of non-coding RNAs in tumors [166]. EIF4A3, an RNA helicase and a core component of the exon junction complex, plays crucial roles in splicing and is activated in a variety of cancers and involved in autophagy regulation [166,167]. EIF4A3 is also involved in the regulation of angiogenesis [156]. Thus, EIF4A3 was shown to contribute to LINC00667-dependent stimulation of vascular endothelial cell proliferation and migration by increasing the stability of vascular endothelial growth factor A (VEGFA). LINC00667 is overexpressed in NSCLC and, according to the dual luciferase reporter assay, it cannot bind and regulate VEGFA promoter activity; on the contrary, it can regulate the expression of VEGFA at the post-transcriptional level [156]. StarBase v3.0 scanning revealed 71 RBPs capable of binding to both LINC00667 and VEGFA mRNA, five (ILF3, MOV10, EIF4A3, ADAR, and IGF2BP2) being overexpressed in NSCLC. Knockdown experiments demonstrated that among these five RBPs, only EIF4A3 could increase the mRNA and protein levels of VEGFA [156] (Table 4). Experiments with actinomycin D showed the ability of EIF4A3 to increase the half-life of VEGFA mRNA in NSCLC. Furthermore, direct binding of EIF4A3 protein to VEGFA mRNA and lncRNA LINC00667 was validated using RIP and RNA pull-down assays. In summary, LINC00667 in the RNP complex containing EIF4A3 RBP and VEGFA mRNA can promote the stabilization of VEGFA mRNA, proliferation, migration, and neoangiogenesis in NSCLC [156].

Most relapses after surgery in patients with NSCLC indicate the presence of persisting cancer stem cells (CSCs). CSC progression is associated with the Hippo pathway [168,169]. The upregulated Hippo pathway kinase LATS1/2 (large tumor suppressor kinase 1, 2) can phosphorylate and inactivate YAP and TAZ. Several other lncRNAs contribute to maintaining the stemness in NSCLC. For instance, the antisense lncRNA MACC1-AS1 is a homolog of the last intron of the metastasis associated in colon cancer 1 (MACC1) gene. MACC1-AS1 exhibits carcinogenic properties and is upregulated in NSCLC [157]. Its overexpression inhibited YAP and TAZ, inactivated the Hippo pathway, and enhanced the stemness of NSCLC cells via downregulating the expression of LATS1/2 kinase at the mRNA level.

Up-frameshift protein 1 (UPF1) is an evolutionarily conserved protein with RNA/DNA-dependent ATPase and RNA helicase activity. UPF1 can reduce mRNA stability; in particular, it can repress the stability of suppressive LATS1/2 kinase mRNA. Direct binding of UPF1 to both MACC1-AS1 and LATS1/2 mRNA was demonstrated by RIP analysis using the RNA-Binding Protein Immunoprecipitation Kit [157]. Overall, MACC1-AS1 in complex with UPF1 RBP can drive NSCLC cell stemness via inhibiting the Hippo pathway LATS1/2 kinase (Table 4).

Many known RBP m^6^A readers contain an YTH domain that specifically recognizes m^6^A over A. They belong to the YTHDF1/2/3 family and are predominantly localized in the cytoplasm [170]. m^6^A-modified lncRNA DLGAP1 (disks large-associated protein 1) antisense RNA 2 (DLGAP1-AS2) was shown to play an important role in NSCLC [159]. METTL3 m^6^A-methyltransferase enhanced its stability via m^6^A transfer to DLGAP1-AS2. At the same time, YTHDF1, the m^6^A-reader RBP that functions as a part of the RNP complex, was shown to transfer m^6^A to c-Myc mRNA, increasing its stability [159]. This mechanism mediates DLGAP1-AS2-dependent oncogenesis and NSCLC progression, stimulation of c-Myc-dependent aerobic glycolysis, and deteriorated prognosis of patients (Table 4).

#### 3.2.5. LncRNAs Mediated by Both miRNA and RBP

**Figure 2:**
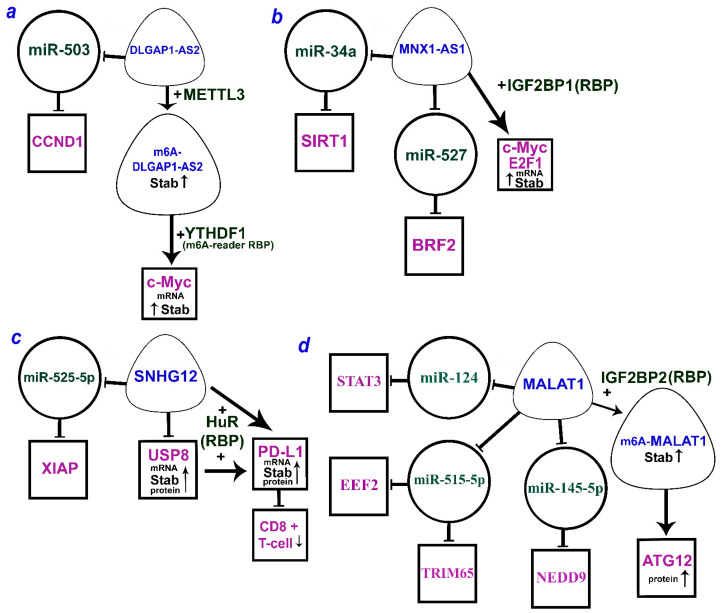
The multiple mechanisms of lncRNAs (**a**) DLGAP1-AS2 (DLGAP1 antisense RNA 2), (**b**) MNX1-AS1 (MNX1 antisense RNA 1), (**c**) SNHG12 (small nucleolar RNA host gene 12), and (**d**) MALAT1 (metastasis-associated lung adenocarcinoma transcript 1) by ceRNA model and through the RBP (according to the following works [33,63,64,65,66,88,146,147,151,159]). LncRNAs are within the soft triangles; miRNAs are within the circles; mRNA/proteins are within the squares; blunt arrows indicate inhibitory interactions; a straight arrow indicates activation of targets.

It is worth noting that there are a number of lncRNAs that can function by both the ceRNA mechanism and alternative mechanisms. Figure 2a–c shows three lncRNAs (DLGAP1-AS2, MNX1-AS1, and SNHG12) that can act via both miRNA and RBP. For the extensively studied MALAT1, ATG12 upregulation was shown to involve RBP IGF2BP2 as well as different axes according to the ceRNA model (Figure 2d). Moreover, IGF2BP2 stabilizes MALAT1 due to m^6^A methylation.

Comparing the data from Section 2 and Section 3 implies that the oncogenic lncRNAs dominate both among lncRNAs that function via ceRNA and lncRNAs utilizing alternative mechanisms. Otherwise, it is possible that suppressor lncRNAs are less studied.

Summarizing Section 3, lncRNAs can significantly affect both the level and stability of a target mRNA and translation rate. Moreover, the mechanisms mediated by the binding of RNA to RNA, or DNA (with a promoter site) as well as by lncRNA interactions with mRNA through a protein mediator, RBP or the RBP variant hnRNP, were revealed. To date, 12 RBPs are known to cooperate with lncRNA in NSCLC: IGF2BP1/2/3, HuR/ELAVL1, FBL, EIF4A3, UPF1, WDR5, YTHDF1, and three hnRNPs with RBP function, namely hnRNPD (AUF1), hnRNPK, and hnRNPU. All 12 RBPs affected the level as well as stability of a target mRNA. RBP IGF2BP2 and YTHDF1 were also involved in m^6^A transfer to CDC6 mRNA or to DLGAP1-AS2 lncRNA, which also contributed to their stabilization. Moreover, we noted that four lncRNAs interact with mRNAs mediated by both miRNAs and RBPs.

## 4. Major Signaling Pathways and Networks Involving lncRNAs in NSCLC

As shown in Section 2 and Section 3, there is mounting evidence on the role of lncRNAs in NSCLC. Target protein arrays selected for our comprehensive review (Table 1, Table 2, Table 3 and Table 4) were analyzed using the DAVID database to identify pathways overrepresented by KEGG, Wikipathways, and the Reactome pathway.

Since a number of lncRNAs appear to play roles in NSCLC, it is important to determine if they affect pathways essential for lung cancer development. lncRNA-dependent changes in the expression of protein-coding genes are highly variable, ranging from several tens of a percent to several orders of magnitude (see, for example [171]). Some processes, such as cell cycle regulation, are sensitive to even the slightest changes in the levels of certain proteins, while others respond only to significantly larger changes. Moreover, if a lncRNA affects several proteins involved in the same pathway, it can result in a significant impact signaling pathway or, conversely, mitigate the change. Investigating these effects is necessary to better understand the potential use of lncRNAs in clinical practice.

We selected a set of target proteins interacting with lncRNAs via the ceRNA mechanism (Table 1 and Table 2). We separately analyzed the overrepresented signaling pathways for it. Since a smaller amount of lncRNA affects protein expression in NSCLC through various alternative mechanisms (Table 3 and Table 4), we analyzed all these mechanisms in total, both separately and together with previously processed data.

In agreement with the published data, lncRNAs acting via the sponge mechanism affect the following proteins, crucial for the signaling in NSCLC according to the KEGG database: ERBB2, GRB2, PDK1, CCND1, CDK1, E2F3, BAX, and STAT3 (https://www.kegg.jp/pathway/hsa05223, accessed on 19 June 2023).

Alternative mechanisms mediating the influence of lncRNA on expression involve CCND1, AKT1, and STAT3 (https://www.kegg.jp/pathway/hsa05223, accessed on 19 June 2023).

It should be noted that according to the KEGG scheme, none of the target proteins for each mechanism of action was assigned to the most significant oncogenes or oncosuppressors.

Undoubtedly, proteins that are not included in the KEGG scheme, can also influence carcinogenesis and have potential applications in clinical practice. According to the KEGG_pathway database (evaluated with https://david.ncifcrf.gov/, accessed on 19 June 2023), 25 out of 107 proteins regulated by the endogenous competition and 13 out of 45 proteins regulated by alternative lncRNA-dependent mechanisms fall into the “Pathways in cancer” section. One example of these proteins is the tumor suppressor PTEN downregulated in NSCLC lncRNA AC078883.3 via miR-19a binding.

Several lncRNA-regulated proteins overlap with the set of proteins encoded by genes with mutations or methylation patterns associated with alterations in gene expression typical of lung adenocarcinoma and lung squamous cell carcinoma [172]. LncRNAs affect the expression of ERBB2, ROS, PTEN, MMD2, CCND1, and CDK4 via competition for binding to miRNAs that target the mRNAs of the listed genes. The expression of AKT1 and CCND1 is regulated by alternative mechanisms mediating the interactions with lncRNAs. It should be noted that the most frequently mutated genes in NSCLC (TP53 and CDKN2A) are presumably not affected by lncRNAs.

Proteins regulated by lncRNAs via the sponge mechanism (ceRNA) are significantly overrepresented in the following signaling pathways: JAK-STAT (8 proteins), cell cycle (14), p53 (11) according to KEGG_pathway; VEGFA-VEGFR2 (12), cell cycle (14), DNA damage response (only ATM dependent) (12) according to Wikipathways; transcriptional regulation by TP53 (13), interleukin-4 and interleukin-13 signaling (11), signaling by interleukin (15), cytokine signaling immune system (17), immune system (24) according to Reactome pathway (evaluated with https://david.ncifcrf.gov/, accessed on 19 June 2023).

Proteins regulated by lncRNAs via alternative mechanisms are significantly overrepresented in the following signaling pathways: chemokine signaling pathway (6), cytokine–cytokine receptor interaction (5), Hippo (8) according to KEGG_pathway; processes (6), prostaglandin signaling (3), IL-7 signaling pathway (5) according to Wikipathways; and interleukin-4 and interleukin-13 signaling (8) according to Reactome pathway (evaluated with https://david.ncifcrf.gov/, accessed on 19 June 2023).

**Figure 3:**
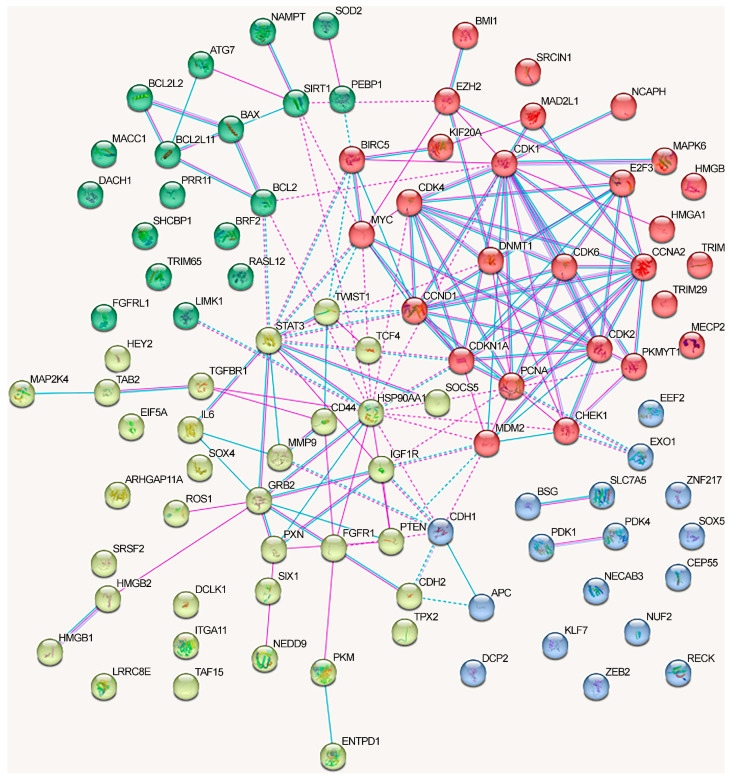
Associations between proteins regulated in NSCLC by lncRNAs using the ceRNA mechanism (taken from Table 1 and Table 2). Depicted according to the scheme from the STRING database. Experimental data are indicated by pink lines, data from databases are shown by blue lines. Bonds between proteins from the same cluster are indicated by a solid line, and those between proteins from different clusters are shown by a dotted line.

We analyzed the interactions between proteins regulated by lncRNAs in NSCLC using the STRING database (Figure 3). We included only the experimentally confirmed data (positions experiment (pink lines) and databases (blue lines)). First, we analyzed the proteins whose genes expression is regulated by lncRNAs via the ceRNA mechanism. As can be seen, at least some proteins have multiple connections (Figure 3). The network can be divided into four clusters, three of which have a fairly high degree of connectivity.

For a separate analysis of each cluster, we included only those proteins that reliably interacted with any protein in the same cluster and searched for the overrepresented pathways within the cluster. The first cluster (in red) primarily included the proteins associated with the cell cycle (KEGG_pathway (13 of 21), Wikipathways (12), Reactome pathway (16)), including the G1/S checkpoint (Wikipathways (9)), mitotic G1 phase and G1/S transition (Reactome pathway (9)), DNA damage response (Wikipathways (9)), and p53 signaling pathway (KEGG_pathway (8)) (evaluated with https://david.ncifcrf.gov/, accessed on 19 June 2023) (Figure 3).

Proteins from the second cluster (highlighted in yellow) were associated with signaling by interleukins (Reactome pathway (10 of 24)), interleukin-4 and interleukin-13 signaling (Reactome pathway (6)), IL6 signaling pathway (Wikipathways (4)), and cytokine signaling in the immune system (Reactome pathway (11)) (evaluated with https://david.ncifcrf.gov/, accessed on 19 June 2023). The third cluster (in green) mainly included proteins associated with the regulation of apoptosis and autophagy (Figure 3).

**Figure 4:**
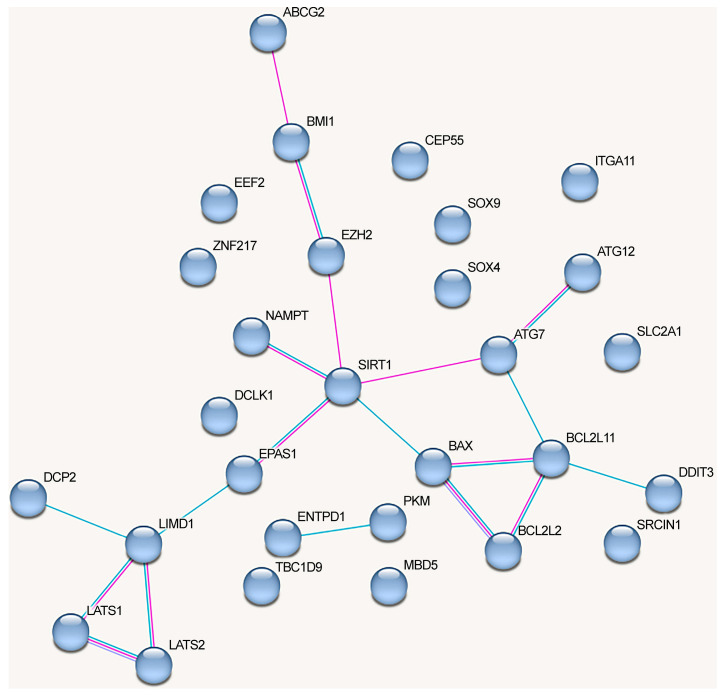
Associations between proteins regulated in NSCLC by lncRNAs in a mechanism different from that of ceRNAs (taken from Table 3 and Table 4). Depicted according to the scheme from the STRING database. Experimental data are indicated by pink lines, data from databases are shown by blue lines.

The network of protein interactions regulated by the alternative lncRNA-dependent mechanisms demonstrates a lack of cohesion and cannot be reliably clusterized (Figure 4). These proteins are involved in the following signaling pathways: cellular responses to stress (Reactome pathway (8 of 18)), signaling by interleukins (Reactome pathway (8)), interleukin-4 and interleukin-13 signaling (Reactome pathway (8)), IL7 signaling pathway (Wikipathways (5)), neovascularization processes (Wikipathways (6)), Hippo (KEGG_pathway (7)), and Notch signaling pathway (Wikipathways (5)) (evaluated with https://david.ncifcrf.gov/, accessed on 19 June 2023) (Figure 4).

**Figure 5:**
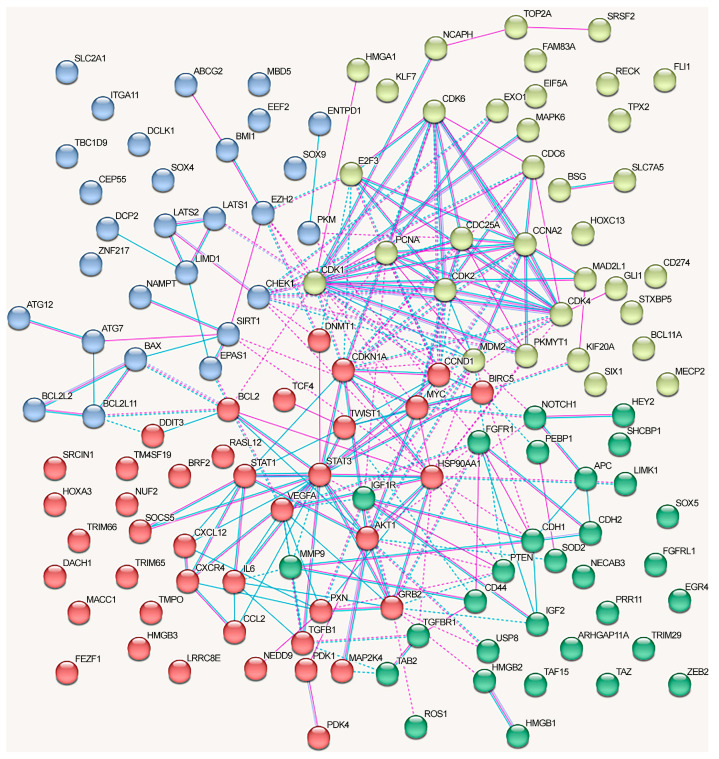
Associations among all known proteins regulated by lncRNAs in NSCLC (taken from Table 1, Table 2, Table 3 and Table 4). Depicted according to the scheme from the STRING database. Experimental data are indicated by pink lines, data from databases are shown by blue lines. Bonds between proteins from the same cluster are indicated by a solid line, and those between proteins from different clusters are shown by a dotted line.

The graph containing all proteins affected by lncRNAs can be divided into clusters, although there are some obvious differences from the network in Figure 3 (Figure 5).

The proteins from the first cluster (yellow color) are associated with cell cycle checkpoints (Reactome pathway (9 of 20)) and TP53-regulated transcription of cell cycle genes (Reactome pathway (4)) (evaluated with https://david.ncifcrf.gov/, accessed on 19 June 2023). The second protein cluster (red color) is related to cytokine signaling in the immune system (Reactome pathway (17/25)), VEGFA-VEGFR2 signaling (Wikipathways (12)), and focal adhesion (KEGG_pathway, Wikipathways (6)) signaling pathways (evaluated with https://david.ncifcrf.gov/, accessed on 19 June 2023). The third cluster (blue color) did not exhibit any significant trends, although three proteins that belonged to the cluster were associated with the Hippo signaling pathway (KEGG_pathway) (evaluated with https://david.ncifcrf.gov/, accessed on 19 June 2023). The fourth cluster (green color) was associated with the Hippo signaling pathway, including Hippo–Merlin signaling dysregulation (Wikipathways 5/12) and Hippo signaling regulation (4) (evaluated with https://david.ncifcrf.gov/, accessed on 19 June 2023) (Figure 5).

Thus, we can assume that:

1. The oncogenic and oncosuppressive proteins crucial for NSCLC development regulated by lncRNA only moderately overlapped with the major proteins from the NSCLC-related signaling pathways.
2. The oncogenic and oncosuppressive proteins crucial for NSCLC development affected by lncRNA only moderately overlap with the proteins encoded by genes most frequently mutated or undergoing methylation changes in NSCLC.
3. Among the oncogenic and oncosuppressive proteins regulated by lncRNAs via the ceRNA mechanism, the overrepresented proteins are associated with the regulation of cell cycle and DNA damage response, the cytokine and immune systems, and the JAK-STAT and VEGFA-VEGFR2 signaling pathways.
4. Among the oncogenic and oncosuppressive proteins regulated through lncRNAs via alternative mechanisms, the overrepresented proteins are associated with the cytokine system, the Hippo signaling pathway, and neovascularization.
5. The effects of different lncRNAs on NSCLC are potentially cumulative since the affected proteins, jointly involved in such processes as cell cycle regulation, cytokine system, and the Hippo signaling pathway, may directly interact with each other.

## 5. Conclusions

In conclusion, lncRNAs contribute to regulating the target genes in NSCLC through several established mechanisms. As data on interactomes mediated by competing endogenous RNAs in NSCLC have been extensively discussed in recent reviews [1,2,20], we briefly presented lncRNA/miRNA/mRNA axes identified in the last 5 years and their functions in the NSCLC pathogenesis in Table 1 and Table 2. In addition, we used the mRNA targets of these axes in the bioinformatics prediction of signaling pathways.

In contrast, we focused on the multiaxial lncRNAs: tumor suppressor GHRLOS and oncogenic LINC01426, MALAT1, TYMSOS, and XIST. We covered in greater detail the cascades they regulate, since this phenomenon demonstrates the multiplicity of lncRNA functions and their potential ability to dynamically switch the regulation of alternative targets depending on the other changes in an NSCLC cell or tissue.

Alternative mechanisms are discussed in nuance. Particular attention was paid to RBPs, which have specific domains, possess high plasticity, and can interact directly with both messenger RNAs and non-coding RNAs, as well as with other proteins, to form triple complexes. Of note, the ability of RBPs to regulate mRNAs at the post-transcriptional level was reported in [19,20,173], and recent reviews have discussed the cross-functional interactions of lncRNAs with RBPs in cancer and the possible clinical significance of the lncRNA-RBP interaction network [22,174,175]. However, to the best of our knowledge this is the first comprehensive systematic review addressing the mechanisms of cancer-related gene regulation involving the combination of lncRNAs with RBPs in NSCLC or other cancers. We believe that our overview on the alternative lncRNA-dependent regulatory mechanisms in NSCLC will be original and interesting.

Thus, we noticed that lncRNAs could affect both the level and stability of target mRNAs and their translation rate. Moreover, we described RNA binding to RNAs or DNAs (promoter sites) as well as the interactions of lncRNAs with mRNAs through protein mediators, RBP or the RBP variants, namely heterogeneous nuclear ribonucleoprotein (hnRNP). All 12 RBPs involved in NSCLC (IGF2BP1/2/3, HuR, FBL, EIF4A3, WDR5, YTHDF1, hnRNPD, hnRNPK, and hnRNPU) contributed to the stability of target mRNAs. In addition, IGF2BP2 and YTHDF1 were involved in RNA methylation via m^6^A transfer or as m^6^A readers.

Interestingly, several lncRNAs were shown to regulate mRNAs, both via the ceRNA model and alternative mechanisms. The action of four lncRNAs (DLGAP1-AS2, MALAT1, MNX1-AS1, and SNHG12) could be mediated by both miRNAs and RBPs. Thus, once again, the poly-functionality of lncRNAs by describing their regulatory functions in NSCLC pathogenesis is demonstrated.

In the last section, we analyzed protein sets selected for our comprehensive review using the DAVID database to identify the overrepresented pathways according to KEGG, Wikipathways, and the Reactome pathway. Moreover, the proteins regulated by lncRNAs via ceRNA and alternative mechanisms were separately analyzed. Utilizing the STRING website, we evaluated the interactions between target proteins and created the protein networks.

The currently known lncRNA targets appear to be involved only in certain processes and signaling pathways crucial for NSCLC development. These processes are essential for intracellular signaling and include cell cycle regulation, cytokine pathways, JAK-STAT and Hippo signaling pathways, the VEGFA-VEGFR2 pathway, and neovascularization. Furthermore, these lncRNAs simultaneously affect multiple proteins involved in these processes and are interconnected through the interaction network.

## Figures and Tables

**Table 1:** Axes of oncogenic lncRNAs acting via the ceRNA model in NSCLC progression.

lncRNA/miRNA/mRNA Axes	Regulated Processes and Signaling Pathways; Role in Cancer Prognosis, Survival, and Drug Resistance	Ref.
**AC078883.3**/miR-19a/PTEN	Sensitivity to cisplatin	[25]
**ATP2B1**/miR-222-5p/TAB2	Associated with survival and chemosensitivity	[26]
**BCYRN1**/miR-149/PKM2	Cell glycolysis, proliferation, and invasion	[27]
**CCAT1**/miR-152	Promoted cell proliferation, cell invasion, and EMT of NSCLC cell lines and metastasis	[28]
**CCAT1**/miR-490		[29]
**DANCR**/miR-1225-3p/ErbB2	NSCLC cell migration and invasion; enhanced growth and metastasis, larger tumor size, advanced TNM stage, lymph node metastasis, predicted poor prognoses	[30]
**DANCR**/miR-138/Sox4		[31]
**DGCR5**/miR-330-5p/CD44	Promoted lung cancer progression	[32]
**DLGAP1-AS2**/miR-503/cyclin D1	Increased cell proliferation	[33]
**DLX6-AS1**/miR-144/PRR11	Promoted cell proliferation, migration, invasion, EMT, and inhibited apoptosis	[34]
**DLX6-AS1**/miR-16-5p/BMI1		[35]
**DNAH17-AS1**/miR-877-5p/CCNA2	Proliferation, migration, invasion of H1299 and 95D cell lines, and inhibition of apoptosis	[36]
**FEZF1-AS1**/miR-516b-5p/ITGA11	Cell proliferation and migration, preventing cell cycle arrest at the G2/M phase	[37]
**FGD5-AS1**/miR-107/FGFRL1	Increased the proliferation, viability, migration, and invasion of NSCLC cells	[38]
**FGD5-AS1**/miR-944/MACC1		[39]
**FOXD2-AS1**/miR185-5p/SIX1	Promoted colony formation, cell proliferation, migration, invasion, and drug resistance	[40]
**FOXD3-AS1**/miR-127-3p/MDM2	Promoted drug resistance in NSCLC cells	[41]
**H19**/miR-17/STAT3	Promoted growth, migration, and invasion	[42]
**HCP5**/miR-320/survivin	Predicted poor survival	[43]
**HNF1AS1**/miR-92a-3p/MAP2K4	Enhanced proliferation, inhibited apoptosis, and reduced radiotherapy sensitivity	[44]
**HOTAIR**/miR-217/DACH1	Promoted cell migration, invasion, and proliferation	[45]
**HOTTIP**/miR-615-3p/HMGB3	Hypoxia-induced glycolysis	[46]
**HOXA11-AS**/miR-148a-3p/DNMT1	Increasing cell proliferation and inhibition of cell apoptosis	[47]
**HOXD-AS1**/miR-133a/MMP-9	Proliferation, migration, and invasion of NSCLC cells	[48]
**HUWE1**/miR-222-5p/TAB2	Associated with survival and chemosensitivity	[26]
**KCNQ1OT1**/miR-27b-3p/HSP90AA1	Proliferation, migration, and invasion of H460 cells	[49]
**LINC00152**/miR-16-5p/BCL2L2	The migration and invasion ability of NSCLC cells and inhibition of apoptosis	[50]
**LINC00243**/miR-507/PDK4	Proliferation and glycolysis of NSCLC cells	[51]
**LINC00324**/miR-139-5p/IGF1R	Promoted cell proliferation and invasion	[52]
**LINC00511**/miR-625/LRRC8E	Increase in cell proliferation, invasion, and migration, postoperative distant recurrence growth, IC50 value, and metastasis in DDP resistance	[53]
**LINC00511**/miR-98-5p/TGFBR1		[54]
**LINC00518**/miR-185-3p/MECP2	Promoted cell growth by regulating the cell cycle	[55]
STAT3/**LINC00668**/miR-193a/KLF7	NSCLC cell proliferation, migration, invasion, and inhibition of apoptosis	[56]
**LINC01123**/miR-199a-5p/c-Myc	Promoted NSCLC cell proliferation and aerobic glycolysis	[57]
**LINC01207**/miR-525-5p/ARHGAP11A	Proliferation, migration, invasion of cancer cells, and inhibition of cell apoptosis	[58]
**LINC01296**/miR-598/Twist1	Accelerated proliferation, inhibited apoptosis in vitro, and promoted tumor growth in vivo	[59]
**LINC01426**/hsa-miR-143-3P/CDK1	Progression and development of NSCLC	[60]
**LINC01426**/hsa-miR-143-3P/MAD2L1		
**LINC01426**/hsa-miR-3065-3p/TPX2		
**LINC01426**/hsa-miR-508-3p/CHEK1		
**LINC01426**/hsa-miR-508-3p/SHCBP1		
**LINC01426**/hsa-miR-508-3p/EXO1		
**LINC01748**/miR-520a-5p/HMGA1	Cell proliferation, migration, and invasion, inhibition of cell apoptosis in vitro, and increased tumor growth in vivo	[61]
**LOC285758**/miRNA-204/CDK6	Promoted cell survival, migration and invasion	[62]
ERβ/**MALAT1**/miR-515-5p/EEF2	High levels of axosomal MALAT1 increase cell proliferation, migration, invasion, colony formation, inhibit cell apoptosis in vitro, increase tumor growth in vivo in NSCLC	[63]
ERβ/**MALAT1**/miR-515-3p/TRIM65		[64]
ERβ/**MALAT1**/miR145-5p/NEDD9		[65]
ERβ/**MALAT1**/miR-124/STAT3		[66]
**MAPKAPK5-AS1**/miR-490-3p/HMGB2	Promoted proliferation and EMT and induced apoptosis	[67]
**MCM3AP-AS1**/miR-195-5p/E2F3	Proliferation, migration, and invasion of NSCLC cells	[68]
**MEG8**/miR-107/CDK6	Cell proliferation, migration, and invasion	[69]
**MFI2-AS1**/miR-107/NFAT5	Mediated proliferation, migration, invasion, angiogenesis, and metastasis	[70]
**MINCR**/miR-126/SLC7A5	NSCLC cell proliferation, migration, and inhibition of cell apoptosis	[71]
**MIR9-3HG**/miR-138-5p/LIMK1	Promoted proliferation, migration, invasion, EMT, inhibited cell apoptosis in lung squamous cell carcinoma	[72]
**MIR9-3HG**/miR-138-5p/TAF15		
**MNX1-AS1**/miR-34a/SIRT1	Promoted proliferation, migration, invasion, lymph node metastasis, and poor prognosis	[73]
**MNX1-AS1**/miR-527/BRF2		[74]
**MRUL**/miR-17-5p/SRSF2	Promoted cell proliferation, migration, and invasion and is correlated with poor prognosis	[75]
**NCK1-AS1**/miR-512-5p/p21	Shorter overall survival time and faster progression	[76]
**NEAT1**/hsa-mir-98-5p/MAPK6	Progression of NSCLC cells (growth, migration, invasion)	[77]
**OGFRP1**/miR-4640-5p/eIF5A	Promoted proliferation, migration, and invasion	[78]
**PCAT7**/miR-486-5p/CDK4	Promoted the development of NSCLC	[79]
**PKMYT1AR**/miR-485-5p/PKMYT1	Promoted cancer stem cells, tumor cell proliferation, migration, and xenograft tumor formation	[80]
**PRNCR1**/miR-488/HEY2	Cell proliferation, migration, and invasion, and EMT	[81]
**PTPRG-AS1**/miR-200c-3p/TCF4	Promoted viability and enhanced radioresistance	[82]
**PVT1**/miR-551b/FGFR1	Promoted proliferation, migration, and invasion	[83]
**PVT1**/miR-760/IL-6		[84]
E2F1/**SBF2-AS1**/miR-362-3p/GRB2	Tumor growth in vivo and cell proliferation, migration, and invasion in vitro	[85]
**SNHG1**/miR-330-5p/DCLK1	Progression and chemoresistance of NSCLC	[86]
**SNHG11**/miR-485-5p/BSG	Promoted growth, migration, and EMT	[87]
**SNHG12**/miR-525-5p/XIAP	Promoted proliferation and enhanced DDP resistance	[88]
**SNHG14**/miR-34a/HMGB1	Migration, invasion, and inhibition of apoptosis	[89]
**SNHG15**/miR-211-3p/ZNF217	Proliferation and migration of NSCLC cells	[90]
**TATDN1**/miR-451/TRIM66	Promoted cell proliferation and inhibited cell apoptosis	[91]
**TMEM132D-AS1**/miR-766-5p/ENTPD1	Increased cell proliferation	[92]
**TP73-AS1**/miR-34a-5p/TRIM29	Cell proliferation, migration, invasion, tumor growth, cycle progression, cisplatin resistance, and inhibition of apoptosis	[93]
**TP73-AS1**/miR-449a/EZH2		[94]
**TYMSOS**/hsa-miR-195-5p/CHEK1	Progression and development of NSCLC	[60]
**TYMSOS**/hsa-miR-221-3p/KIF20A		
**TYMSOS**/hsa-miR-486-3p/NUF2		
**TYMSOS**/hsa-miR-101-3p/CEP55		
**UCC**/miR-143-3p/SOX5	Promoted EMT	[95]
**VPS9D1-AS1**/hsa-miR-548p/NCAPH	Progression and development of NSCLC	[60]
**WFDC21P**/MIR4293/DCP2	Promoted tumor cell proliferation and metastasis but suppressed apoptosis	[96]
**WT1-AS**/miR-206/NAMPT	Associated with shortened survival	[97]
**XIST**/miR-17/ATG7	Promoted cell proliferation, cell viability, migration, and invasion, inhibited apoptosis, increased autophagy, TGF-β-induced EMT, and pulmonary metastasis of NSCLC	[98]
**XIST**/miR-520/BAX		[99]
**XIST**/miR-137/PXN		[100]
**XIST**/miR-335/SOD2/ROS		[101]
**XIST**/miR-367/miR-141/ZEB2		[102]

Note: EMT—epithelial–mesenchymal transition; DDP—cis-diamminedichloroplatinum(II); LINC01426—long intergenic non-protein coding RNA 1426; MALAT1—metastasis-associated lung adenocarcinoma transcript 1; TYMSOS—TYMS opposite strand RNA; XIST—X-inactive specific transcript; TGF-β—transforming growth factor-β.

**Table 2:** Axes of oncosuppressive lncRNAs acting via the ceRNA model in NSCLC progression.

lncRNA/miRNA/mRNA Axes	Regulated Processes and Signaling Pathways; Role in Cancer Prognosis, Survival, and Drug Resistance	Ref.
**FOXD3-AS1**/miR-150/SRCIN1	Inhibited the proliferation and invasion of H1299 cell lines	[103]
**GAN1**/miR-26a-5p/PTEN	Suppressed cell proliferation, colony formation, and cell cycle progression and induced apoptosis	[104]
**GATA6-AS1**/miR-543/RKIP	Inhibited proliferation, migration, invasion, and EMT of NSCLC cells	[105]
**TP53**/**GHRLOS**/miR-346/APC	Suppressed cancer cell proliferation and invasion and promoted cell apoptosis	[106]
**TP53**/**GHRLOS**/miR-346/Bax		
**TP53**/**GHRLOS**/miR-346/Bcl-2		
**TP53**/**GHRLOS**/miR-346/CDK2		
**TP53**/**GHRLOS**/miR-346/E-cadherin		
**TP53**/**GHRLOS**/miR-346/N-cadherin		
**TP53**/**GHRLOS**/miR-346/PCNA		
**HCG11**/miR-522-3p/SOCS5	Inhibition of cell viability, migration, and invasion	[107]
**c-Myc**/**LINC00173**/miR-1275/PROCA1, ZFP36L2, and BCL2	Cisplatin chemosensitivity, apoptosis	[108]
**LINC01128**/miR-25-3p/PTEN	Promoted EGFR-TKI resistance	[109]
**LINC00494**/miR-150-3p/SRCIN1	Inhibited NSCLC cell proliferation, tumor growth in vivo	[110]
**MAGI2-AS3**/miR-25/RECK	Decreased NSCLC cell invasion and migration	[111]
**MT1JP**/miRNA-423-3p/Bim	Suppressed cell proliferation and increased cell apoptosis	[112]
**SOX2-OT**/miR-30d-5p/PDK1	Could inhibit the proliferation, migration, and invasion of NSCLC cells and promote cell apoptosis	[113]
**TP53TG1**/miR-18a/PTEN	Cisplatin sensitivity and apoptosis of A549/DDP cells	[114]
**TPTEP1**/miR-328-5p/SRCIN1	Inhibited cell proliferation and induced apoptosis	[115]

Note: EMT—epithelial–mesenchymal transition; DDP—cis-diamminedichloroplatinum(II); FOXD3-AS1—forkhead box D3 antisense RNA 1; GHRLOS—ghrelin opposite strand/antisense RNA; EGFR-TKI resistance—epidermal growth factor receptor tyrosine kinase inhibitors resistance.

**Table 4:** Action of lncRNAs mediated by RNA-binding proteins (RBPs) in NSCLC progression.

Mechanisms, Axes	LncRNAs/Axes in Processes, Pathways, Prognosis, Survival, and Drug Resistance	Ref.
**IGF2BP1/2/3 as an RNA-binding protein (RBP)**		
**LCAT1**↑→m^6^A-IGF2BP2(RBP)↑stab →m^6^A-CDC6↑mRNAstab	Promotes NSCLC cell growth, migration, and poor patient survival	[143]
**Linc-SPRY3-2/3/4**↓ov-ex+IGF2BP3(RBP) /HMGA2, c-MYC↓mRNAstab	Suppresses NSCLC and enhances cell radiation response	[144]
FOXP3→(pr)**LINC01232**↑+IGF2BP2(RBP)→TGFBR1↑stab	Promotes TGF-β signaling, NSCLC cell stemness	[145]
IGF2BP2(RBP)↑→m^6^A-**MALAT1**↑stab→ATG12↑protein	Promotes NSCLC proliferation; reduces OS, DFS	[146]
c-Myc→**MNX1-AS1**↑+IGF2BP1(RBP) ↔c-Myc, E2F1↑mRNAstab→c-Myc-sign	Promotes cell cycle progression, proliferation in vitro, in vivo; poor clinical outcomes	[147]
**lnc-THOR**↑→IGF2BP1(RBP)↑ →IGF2, Gli1, Myc, SOX9↑mRNAstab	Enhances NSCLC cell proliferation, migration, and invasion	[148]
**HuR/ELAVL1 as an RNA-binding protein (RBP)**		
**FENDRR**↓ov-ex/MDR1↓mRNAstab ↔MDR1↑3’UTR← HuR(RBP)	Suppresses NSCLC cell stemness	[149]
E2F1→**MCF2L-AS1**↑+HuR(RBP)→CCND1↑mRNAstab	Drives NSCLC cell growth and induces gefitinib resistance	[150]
**SNHG12**↑+HuR(RBP)→USP8↑mRNAstab,protein →PD-L1↑mRNAstab, protein↑/CD8+T-cell↓	Promotes proliferation, migration, invasion, and immune escape in vitro/in vivo	[151]
**Heterogeneous nuclear ribonucleoproteins (hnRNPs) with RBP function**		
**SChLAP1**↑/hnRNPD(AUF1, RBP)↓/PDL1↑mRNAstab	Enhances proliferation, immune evasion	[152]
DNA-meth/**DIO3OS**↓ov-ex/hnRNPK↓ /MYC,DNA,mRNA↓/CDC25A↓	Ectopic expression of DIO3OS suppresses NSCLC tumorigenesis, metastasis in vivo	[153]
**LIMD1-AS1**↓ov-ex+hnRNPU→LIMD1↑mRNAstab	Suppresses NSCLC progression	[154]
**Other RNA-binding proteins (RBP) as mediators of lncRNAs**		
**FAM83A-AS1**↑+FBL(RBP)→FAM83A↑pre-mRNAstab	Promotes LUAC metastasis, ERK signaling; low OS, PFS	[155]
**LINC00667**↑+EIF4A3(RBP)→VEGFA↑mRNAstab	Promotes proliferation, migration, and angiogenesis	[156]
**MACC1-AS1**+UPF1(RBP)→LATS1/2↓mRNAdestab	Drives NSCLC cell stemness through inhibition of the Hippo pathway	[157]
**TM4SF19-AS1**↑+WDR5(RBP)→TM4SF19(pr-WDR5) →(DNA-demeth-pr) TM4SF19↑mRNA	Facilitates proliferation, adhesion of lung squamous cell carcinoma	[158]
METTL3→m^6^A-**DLGAP1-AS2**↑stab +YTHDF1(m^6^A-reader RBP)→c-Myc↑mRNAstab	Promotes aerobic glycolysis; correlated with advanced stages, poor prognosis	[159]

Note: stab—stability; ov-ex—overexpression; demeth-pr—demethylated promoter; meth—methylation; DFS—disease-free survival; OS—overall survival; PFS—progression-free survival; p-w—pathway; phosph—phosphorylation; sign—signaling; ↑ ↓—increase or decrease in expression or stabilization; →—activation; /—inhibition; ↔—a positive feedback.

## Data Availability

Not applicable.

## References

[1] Ginn L., Shi L., Montagna M., Garofalo M. (2020). LncRNAs in Non-Small-Cell Lung Cancer. Non-Coding RNA.

[2] Zhao M., Feng J., Tang L. (2021). Competing endogenous RNAs in lung cancer. Cancer Biol. Med..

[3] Djebali S., Davis C.A., Merkel A., Dobin A., Lassmann T., Mortazavi A., Tanzer A., Lagarde J., Lin W., Schlesinger F. (2012). Landscape of transcription in human cells. Nature.

[4] Forrest A.R., Kawaji H., Rehli M., Baillie J.K., de Hoon M.J., Haberle V., Lassmann T., Kulakovskiy I.V., Lizio M., FANTOM Consortium and the RIKEN PMI and CLST (DGT) (2014). A promoter-level mammalian expression atlas. Nature.

[5] Ling H., Girnita L., Buda O., Calin G.A. (2017). Non-coding RNAs: The cancer genome dark matter that matters!. Clin. Chem. Lab. Med..

[6] Slack F.J., Chinnaiyan A.M. (2019). The Role of Non-coding RNAs in Oncology. Cell.

[7] Kielbowski K., Ptaszynski K., Wojcik J., Wojtys M.E. (2023). The role of selected non-coding RNAs in the biology of non-small cell lung cancer. Adv. Med. Sci..

[8] Baek D., Villen J., Shin C., Camargo F.D., Gygi S.P., Bartel D.P. (2008). The impact of microRNAs on protein output. Nature.

[9] Bartel D.P. (2009). MicroRNAs: Target recognition and regulatory functions. Cell.

[10] Lee Y.S., Dutta A. (2009). MicroRNAs in cancer. Annu. Rev. Pathol..

[11] Sadakierska-Chudy A. (2020). MicroRNAs: Diverse Mechanisms of Action and Their Potential Applications as Cancer Epi-Therapeutics. Biomolecules.

[12] Gerstein M.B., Kundaje A., Hariharan M., Landt S.G., Yan K.K., Cheng C., Mu X.J., Khurana E., Rozowsky J., Alexander R. (2012). Architecture of the human regulatory network derived from ENCODE data. Nature.

[13] Quinn J.J., Chang H.Y. (2016). Unique features of long non-coding RNA biogenesis and function. Nat. Rev. Genet..

[14] Iyer M.K., Niknafs Y.S., Malik R., Singhal U., Sahu A., Hosono Y., Barrette T.R., Prensner J.R., Evans J.R., Zhao S. (2015). The landscape of long noncoding RNAs in the human transcriptome. Nat. Genet..

[15] Al-Rugeebah A., Alanazi M., Parine N.R. (2019). MEG3: An Oncogenic Long Non-coding RNA in Different Cancers. Pathol. Oncol. Res..

[16] Zhang L., Zhao F., Li W., Song G., Kasim V., Wu S. (2022). The Biological Roles and Molecular Mechanisms of Long Non-Coding RNA MEG3 in the Hallmarks of Cancer. Cancers.

[17] Salmena L., Poliseno L., Tay Y., Kats L., Pandolfi P.P. (2011). A ceRNA hypothesis: The Rosetta Stone of a hidden RNA language?. Cell.

[18] Rajakumar S., Jamespaulraj S., Shah Y., Kejamurthy P., Jaganathan M.K., Mahalingam G., Ramya Devi K.T. (2023). Long non-coding RNAs: An overview on miRNA sponging and its co-regulation in lung cancer. Mol. Biol. Rep..

[19] Dreyfuss G., Kim V.N., Kataoka N. (2002). Messenger-RNA-binding proteins and the messages they carry. Nat. Rev. Mol. Cell Biol..

[20] Mitchell S.F., Parker R. (2014). Principles and properties of eukaryotic mRNPs. Mol. Cell.

[21] Pereira B., Billaud M., Almeida R. (2017). RNA-Binding Proteins in Cancer: Old Players and New Actors. Trends Cancer.

[22] Jonas K., Calin G.A., Pichler M. (2020). RNA-Binding Proteins as Important Regulators of Long Non-Coding RNAs in Cancer. Int. J. Mol. Sci..

[23] Murtha M., Esteller M. (2016). Extraordinary Cancer Epigenomics: Thinking Outside the Classical Coding and Promoter Box. Trends Cancer.

[24] Singh K.P., Gupta S. (2022). 3D Modeling of Non-coding RNA Interactions. Adv. Exp. Med. Biol..

[25] Xing S., Qu Y., Li C., Huang A., Tong S., Wu C., Fan K. (2019). Deregulation of lncRNA-AC078883.3 and microRNA-19a is involved in the development of chemoresistance to cisplatin via modulating signaling pathway of PTEN/AKT. J. Cell Physiol..

[26] Kong X., Hu S., Yuan Y., Du Y., Zhu Z., Song Z., Lu S., Zhao C., Yan D. (2020). Analysis of lncRNA, miRNA and mRNA-associated ceRNA networks and identification of potential drug targets for drug-resistant non-small cell lung cancer. J. Cancer.

[27] Lang N., Wang C., Zhao J., Shi F., Wu T., Cao H. (2020). Long non-coding RNA BCYRN1 promotes glycolysis and tumor progression by regulating the miR-149/PKM2 axis in non-small-cell lung cancer. Mol. Med. Rep..

[28] Li N., Hao W., Yang J., Guo Y., Guo Y., Du Y. (2020). Long non-coding RNA colon cancer-associated transcript-1 regulates tumor cell proliferation and invasion of non-small-cell lung cancer through suppressing miR-152. Geriatr. Gerontol. Int..

[29] Wang J., Sun N., Han W., Tong L., Xu T., Li G. (2021). Long non-coding RNA CCAT1 sponges miR-490 to enhance cell proliferation and migration of non-small cell lung cancer. Thorac. Cancer.

[30] Huang Y.F., Zhang Y., Fu X. (2021). Long non-coding RNA DANCR promoted non-small cell lung cancer cells metastasis via modulating of miR-1225-3p/ErbB2 signal. Eur. Rev. Med. Pharmacol. Sci..

[31] Bai Y., Zhang G., Chu H., Li P., Li J. (2019). The positive feedback loop of lncRNA DANCR/miR-138/Sox4 facilitates malignancy in non-small cell lung cancer. Am. J. Cancer Res..

[32] Wang R., Dong H.X., Zeng J., Pan J., Jin X.Y. (2018). LncRNA DGCR5 contributes to CSC-like properties via modulating miR-330-5p/CD44 in NSCLC. J. Cell Physiol..

[33] Wang L., Tang L., Ge T., Zhu F., Liu D., Guo H., Qian P., Xu N. (2021). LncRNA DLGAP1-AS2 regulates miR-503/cyclin D1 to promote cell proliferation in non-small cell lung cancer. BMC Pulm. Med..

[34] Huang Y., Ni R., Wang J., Liu Y. (2019). Knockdown of lncRNA DLX6-AS1 inhibits cell proliferation, migration and invasion while promotes apoptosis by downregulating PRR11 expression and upregulating miR-144 in non-small cell lung cancer. Biomed. Pharmacother..

[35] Wu C., Lin W., Fu F. (2021). Long non-coding RNA DLX6-AS1 knockdown suppresses the tumorigenesis and progression of non-small cell lung cancer through microRNA-16-5p/BMI1 axis. Transl. Cancer Res..

[36] Du L.J., Mao L.J., Jing R.J. (2020). Long noncoding RNA DNAH17-AS1 promotes tumorigenesis and metastasis of non-small cell lung cancer via regulating miR-877-5p/CCNA2 pathway. Biochem. Biophys. Res. Commun..

[37] Song H., Li H., Ding X., Li M., Shen H., Li Y., Zhang X., Xing L. (2020). Long non-coding RNA FEZF1-AS1 facilitates non-small cell lung cancer progression via the ITGA11/miR-516b-5p axis. Int. J. Oncol..

[38] Fan Y., Li H., Yu Z., Dong W., Cui X., Ma J., Li S. (2020). Long non-coding RNA FGD5-AS1 promotes non-small cell lung cancer cell proliferation through sponging hsa-miR-107 to up-regulate FGFRL1. Biosci. Rep..

[39] Lv J., Li Q., Ma R., Wang Z., Yu Y., Liu H., Miao Y., Jiang S. (2021). Long Noncoding RNA FGD5-AS1 Knockdown Decrease Viability, Migration, and Invasion of Non-Small Cell Lung Cancer (NSCLC) Cells by Regulating the MicroRNA-944/MACC1 Axis. Technol. Cancer Res. Treat..

[40] Ge P., Cao L., Yao Y.J., Jing R.J., Wang W., Li H.J. (2019). lncRNA FOXD2-AS1 confers cisplatin resistance of non-small-cell lung cancer via regulation of miR185-5p-SIX1 axis. OncoTargets Ther..

[41] Zeng Z., Zhao G., Zhu H., Nie L., He L., Liu J., Li R., Xiao S., Hua G. (2020). LncRNA FOXD3-AS1 promoted chemo-resistance of NSCLC cells via directly acting on miR-127-3p/MDM2 axis. Cancer Cell Int..

[42] Huang Z., Lei W., Hu H.B., Zhang H., Zhu Y. (2018). H19 promotes non-small-cell lung cancer (NSCLC) development through STAT3 signaling via sponging miR-17. J. Cell Physiol..

[43] Li C., Lei Z., Peng B., Zhu J., Chen L. (2020). LncRNA HCP5 Stimulates the Proliferation of Non-Small Cell Lung Cancer Cells by Up-Regulating Survivin through the Down-Regulation of miR-320. Cancer Manag. Res..

[44] Wang Z., Liu L., Du Y., Mi Y., Wang L. (2021). The HNF1A-AS1/miR-92a-3p axis affects the radiosensitivity of non-small cell lung cancer by competitively regulating the JNK pathway. Cell Biol. Toxicol..

[45] Chen S.S., Peng M., Zhou G.Z., Pu Y.C., Yi M.C., Zhu Y., Jiang B. (2019). Long non-coding RNA HOTAIR regulates the development of non-small cell lung cancer through miR-217/DACH1 signaling pathway. Eur. Rev. Med. Pharmacol. Sci..

[46] Shi J., Wang H., Feng W., Huang S., An J., Qiu Y., Wu K. (2019). Long non-coding RNA HOTTIP promotes hypoxia-induced glycolysis through targeting miR-615-3p/HMGB3 axis in non-small cell lung cancer cells. Eur. J. Pharmacol..

[47] Bai Y., Lang L., Zhao W., Niu R. (2019). Long Non-Coding RNA HOXA11-AS Promotes Non-Small Cell Lung Cancer Tumorigenesis through microRNA-148a-3p/DNMT1 Regulatory Axis. OncoTargets Ther..

[48] Xia H., Jing H., Li Y., Lv X. (2018). Long noncoding RNA HOXD-AS1 promotes non-small cell lung cancer migration and invasion through regulating miR-133b/MMP9 axis. Biomed. Pharmacother..

[49] Dong Z., Yang P., Qiu X., Liang S., Guan B., Yang H., Li F., Sun L., Liu H., Zou G. (2019). KCNQ1OT1 facilitates progression of non-small-cell lung carcinoma via modulating miRNA-27b-3p/HSP90AA1 axis. J. Cell Physiol..

[50] Hu H., Chen C., Chen F., Sun N. (2022). LINC00152 knockdown suppresses tumorigenesis in non-small cell lung cancer via sponging miR-16-5p. J. Thorac. Dis..

[51] Feng X., Yang S. (2020). Long non-coding RNA LINC00243 promotes proliferation and glycolysis in non-small cell lung cancer cells by positively regulating PDK4 through sponging miR-507. Mol. Cell Biochem..

[52] Zhang M., Lin B., Liu Y., Huang T., Chen M., Lian D., Deng S., Zhuang C. (2020). LINC00324 affects non-small cell lung cancer cell proliferation and invasion through regulation of the miR-139-5p/IGF1R axis. Mol. Cell Biochem..

[53] Liu B., Zhou F., Liu H., Wang Y., Wang J., Ren F., Xu S. (2022). Knockdown of LINC00511 decreased cisplatin resistance in non-small cell lung cancer by elevating miR-625 level to suppress the expression of leucine rich repeat containing eight volume-regulated anion channel subunit E. Hum. Exp. Toxicol..

[54] Li C., Li Z., Yi H., Liu Z. (2022). IncRNA Linc00511 Upregulation Elevates TGFBR1 and Participates in the Postoperative Distant Recurrence of Non-Small-Cell Lung Cancer by Targeting miR-98-5p. Crit. Rev. Eukaryot. Gene Expr..

[55] Han X., Wu J., Zhang Y., Song J., Shi Z., Chang H. (2021). LINC00518 Promotes Cell Proliferation by Regulating the Cell Cycle of Lung Adenocarcinoma through miR-185-3p Targeting MECP2. Front. Oncol..

[56] An Y.X., Shang Y.J., Xu Z.W., Zhang Q.C., Wang Z., Xuan W.X., Zhang X.J. (2019). STAT3-induced long noncoding RNA LINC00668 promotes migration and invasion of non-small cell lung cancer via the miR-193a/KLF7 axis. Biomed. Pharmacother..

[57] Hua Q., Jin M., Mi B., Xu F., Li T., Zhao L., Liu J., Huang G. (2019). LINC01123, a c-Myc-activated long non-coding RNA, promotes proliferation and aerobic glycolysis of non-small cell lung cancer through miR-199a-5p/c-Myc axis. J. Hematol. Oncol..

[58] Zhang B., Jin Z., Zhang H. (2022). LINC01207 promotes the progression of non-small cell lung cancer via regulating ARHGAP11A by sponging miR-525-5p. Cancer Biomark. Sect. A Dis. Markers.

[59] Xu L., Wei B., Hui H., Sun Y., Liu Y., Yu X., Dai J. (2019). Positive feedback loop of lncRNA LINC01296/miR-598/Twist1 promotes non-small cell lung cancer tumorigenesis. J. Cell Physiol..

[60] Ji L., Yang T., Liu M., Li J., Si Q., Wang Y., Liu J., Dai L. (2023). Construction of lncRNA TYMSOS/hsa-miR-101-3p/CEP55 and TYMSOS/hsa-miR-195-5p/CHEK1 Axis in Non-small Cell Lung Cancer. Biochem. Genet..

[61] Tan Y., Xu F., Xu L., Cui J. (2022). Long non-coding RNA LINC01748 exerts carcinogenic effects in non-small cell lung cancer cell lines by regulating the microRNA-520a-5p/HMGA1 axis. Int. J. Mol. Med..

[62] Yu X., Liu D., Wang L., Wang L. (2022). LncRNA LOC285758 Induced Non-Small Cell Lung Cancer Development through Up-Regulating CDK6 by Sponge Adsorption of miRNA-204. Iran. J. Public Health.

[63] Rong F., Liu L., Zou C., Zeng J., Xu Y. (2020). MALAT1 Promotes Cell Tumorigenicity through Regulating miR-515-5p/EEF2 Axis in Non-Small Cell Lung Cancer. Cancer Manag. Res..

[64] Wang Y., Zhang Q. (2020). Long Noncoding RNA MALAT1 Knockdown Inhibits Proliferation, Migration, and Invasion and Promotes Apoptosis in Non-Small-Cell Lung Cancer Cells through Regulating miR-515-3p/TRIM65 Axis. Cancer Biother. Radiopharm..

[65] Yu W., Ding J., He M., Chen Y., Wang R., Han Z., Xing E.Z., Zhang C., Yeh S. (2019). Estrogen receptor beta promotes the vasculogenic mimicry (VM) and cell invasion via altering the lncRNA-MALAT1/miR-145-5p/NEDD9 signals in lung cancer. Oncogene.

[66] Li S., Mei Z., Hu H.B., Zhang X. (2018). The lncRNA MALAT1 contributes to non-small cell lung cancer development via modulating miR-124/STAT3 axis. J. Cell Physiol..

[67] Miao J., Gao Y., Guan W., Yu X., Wang Y., Jiang P., Yang L., Xu L., You W. (2023). High level of LncRNA MAPKAPK5-AS1 predicts poor prognosis and contributes to the malignant proliferation and EMT of non-small cell lung cancer via sponging miR-490-3p from HMGB2. Genes Genomics.

[68] Shen D., Li J., Tao K., Jiang Y. (2021). Long non-coding RNA MCM3AP antisense RNA 1 promotes non-small cell lung cancer progression through targeting microRNA-195-5p. Bioengineered.

[69] Liu Y., Li L., Shang P., Song X. (2020). LncRNA MEG8 promotes tumor progression of non-small cell lung cancer via regulating miR-107/CDK6 axis. Anti-Cancer Drugs.

[70] Xu J., Wang H., Shi B., Li N., Xu G., Yan X., Xu L. (2023). Exosomal MFI2-AS1 sponge miR-107 promotes non-small cell lung cancer progression through NFAT5. Cancer Cell Int..

[71] Wang J., Ding M., Zhu H., Cao Y., Zhao W. (2019). Up-regulation of long noncoding RNA MINCR promotes non-small cell of lung cancer growth by negatively regulating miR-126/SLC7A5 axis. Biochem. Biophys. Res. Commun..

[72] Xiong Y., Yang C., Yang X., Ding C., Wang Q., Zhu H. (2022). LncRNA MIR9-3HG enhances LIMK1 mRNA and protein levels to contribute to the carcinogenesis of lung squamous cell carcinoma via sponging miR-138-5p and recruiting TAF15. Pathol. Res. Pract..

[73] Liu G., Zhang Y., Zhang X., Liu Y., Xu Y., Cui S., Li G., Wang J. (2022). LncRNA MNX1-AS1 contributes to lung adenocarcinoma progression by targeting the miR-34a/SIRT1 axis. Am. J. Transl. Res..

[74] Liu H., Han L., Liu Z., Gao N. (2019). Long noncoding RNA MNX1-AS1 contributes to lung cancer progression through the miR-527/BRF2 pathway. J. Cell Physiol..

[75] Chen Y., Shen T., Ding X., Ma C., Cheng L., Sheng L., Du X. (2020). lncRNA MRUL Suppressed Non-Small Cell Lung Cancer Cells Proliferation and Invasion by Targeting miR-17-5p/SRSF2 Axis. BioMed Res. Int..

[76] Luo X., Zhou J., Quan L., Liang Y., Huang P., Chen F., Liu S. (2020). LncRNA NCK1-AS1 promotes non-small cell lung cancer progression via regulating miR-512-5p/p21 axis. Pathol. Res. Pract..

[77] Wu F., Mo Q., Wan X., Dan J., Hu H. (2019). NEAT1/hsa-mir-98-5p/MAPK6 axis is involved in non-small-cell lung cancer development. J. Cell Biochem..

[78] Liu X., Niu N., Li P., Zhai L., Xiao K., Chen W., Zhuang X. (2021). LncRNA OGFRP1 acts as an oncogene in NSCLC via miR-4640-5p/eIF5A axis. Cancer Cell Int..

[79] Geng W., Qiu M., Zhang D., Li P., Sun G., Zhou X. (2022). LncRNA PCAT7 promotes non-small cell lung cancer progression by activating miR-486-5p/CDK4 axis-mediated cell cycle. Am. J. Transl. Res..

[80] He Y., Jiang X., Duan L., Xiong Q., Yuan Y., Liu P., Jiang L., Shen Q., Zhao S., Yang C. (2021). LncRNA PKMYT1AR promotes cancer stem cell maintenance in non-small cell lung cancer via activating Wnt signaling pathway. Mol. Cancer.

[81] Cheng D., Bao C., Zhang X., Lin X., Huang H., Zhao L. (2018). LncRNA PRNCR1 interacts with HEY2 to abolish miR-448-mediated growth inhibition in non-small cell lung cancer. Biomed. Pharmacother..

[82] Ma Q., Niu R., Huang W., Da L., Tang Y., Jiang D., Xi Y., Zhang C. (2020). Long Noncoding RNA PTPRG Antisense RNA 1 Reduces Radiosensitivity of Nonsmall Cell Lung Cancer Cells Via Regulating MiR-200c-3p/TCF4. Technol. Cancer Res. Treat..

[83] Wang X., Cheng Z., Dai L., Jiang T., Li P., Jia L., Jing X., An L., Liu M., Wu S. (2021). LncRNA PVT1 Facilitates Proliferation, Migration and Invasion of NSCLC Cells via miR-551b/FGFR1 Axis. OncoTargets Ther..

[84] Su X.H., Zhu Y.R., Hou Y.J., Li K., Dong N.H. (2020). PVT1 induces NSCLC cell migration and invasion by regulating IL-6 via sponging miR-760. Mol. Cell Probes.

[85] Wang A., Wang J. (2020). E2F1-Induced Overexpression of Long Noncoding RNA SBF2-AS1 Promotes Non-Small-Cell Lung Cancer Metastasis through Regulating miR-362-3p/GRB2 Axis. DNA Cell Biol..

[86] Ge P., Cao L., Zheng M., Yao Y., Wang W., Chen X. (2021). LncRNA SNHG1 contributes to the cisplatin resistance and progression of NSCLC via miR-330-5p/DCLK1 axis. Exp. Mol. Pathol..

[87] Cheng R., Lu X., Xu C., Zhang F., Zhang G. (2020). SNHG11 contributes to NSCLC cell growth and migration by targeting miR-485-5p/BSG axis. Biomed. Pharmacother..

[88] Tan D., Wang S., Zhang P., Peng C., Wu T. (2023). LncRNA SNHG12 Decreases Non-Small Cell Lung Cancer Cell Sensitivity to Cisplatin by Repressing miR-525-5p and Promoting XIAP. Ann. Clin. Lab. Sci..

[89] Jiao P., Hou J., Yao M., Wu J., Ren G. (2019). SNHG14 silencing suppresses the progression and promotes cisplatin sensitivity in non-small cell lung cancer. Biomed. Pharmacother..

[90] Ma X.R., Xu Y.L., Qian J., Wang Y. (2019). Long non-coding RNA SNHG15 accelerates the progression of non-small cell lung cancer by absorbing miR-211-3p. Eur. Rev. Med. Pharmacol. Sci..

[91] Wang L., Shang X., Feng Q. (2019). LncRNA TATDN1 contributes to the cisplatin resistance of non-small cell lung cancer through TATDN1/miR-451/TRIM66 axis. Cancer Biol. Ther..

[92] Wang N., Zhao Q., Huang Y., Wen C., Li Y., Bao M., Wu L. (2023). Lnc-TMEM132D-AS1 as a potential therapeutic target for acquired resistance to osimertinib in non-small-cell lung cancer. Mol. Omics.

[93] Luo S., Shen M., Chen H., Li W., Chen C. (2020). Long non-coding RNA TP73-AS1 accelerates the progression and cisplatin resistance of non-small cell lung cancer by upregulating the expression of TRIM29 via competitively targeting microRNA-34a-5p. Mol. Med. Rep..

[94] Zhang L., Fang F., He X. (2018). Long noncoding RNA TP73-AS1 promotes non-small cell lung cancer progression by competitively sponging miR-449a/EZH2. Biomed. Pharmacother..

[95] Chen R., Zhang C., Cheng Y., Wang S., Lin H., Zhang H. (2021). LncRNA UCC promotes epithelial-mesenchymal transition via the miR-143-3p/SOX5 axis in non-small-cell lung cancer. Lab. Investig..

[96] Zhang Q., Yan Y.F., Lv Q., Li Y.J., Wang R.R., Sun G.B., Pan L., Hu J.X., Xie N., Zhang C. (2021). miR-4293 upregulates lncRNA WFDC21P by suppressing mRNA-decapping enzyme 2 to promote lung carcinoma proliferation. Cell Death Dis..

[97] Li W., Liu Y., Li Z.J., Shi Y., Deng J., Bai J., Ma L., Zeng X.X., Feng S.S., Ren J.L. (2021). Unravelling the Role of LncRNA WT1-AS/miR-206/NAMPT Axis as Prognostic Biomarkers in Lung Adenocarcinoma. Biomolecules.

[98] Sun W., Zu Y., Fu X., Deng Y. (2017). Knockdown of lncRNA-XIST enhances the chemosensitivity of NSCLC cells via suppression of autophagy. Oncol. Rep..

[99] Liu T.T., Li R., Liu X., Zhou X.J., Huo C., Li J.P., Qu Y.Q. (2021). LncRNA XIST acts as a MicroRNA-520 sponge to regulate the Cisplatin resistance in NSCLC cells by mediating BAX through CeRNA network. Int. J. Med. Sci..

[100] Jiang H., Zhang H., Hu X., Li W. (2018). Knockdown of long non-coding RNA XIST inhibits cell viability and invasion by regulating miR-137/PXN axis in non-small cell lung cancer. Int. J. Biol. Macromol..

[101] Liu J., Yao L., Zhang M., Jiang J., Yang M., Wang Y. (2019). Downregulation of LncRNA-XIST inhibited development of non-small cell lung cancer by activating miR-335/SOD2/ROS signal pathway mediated pyroptotic cell death. Aging.

[102] Li C., Wan L., Liu Z., Xu G., Wang S., Su Z., Zhang Y., Zhang C., Liu X., Lei Z. (2018). Long non-coding RNA XIST promotes TGF-beta-induced epithelial-mesenchymal transition by regulating miR-367/141-ZEB2 axis in non-small-cell lung cancer. Cancer Lett..

[103] Ji T., Zhang Y., Wang Z., Hou Z., Gao X., Zhang X. (2020). FOXD3-AS1 suppresses the progression of non-small cell lung cancer by regulating miR-150/SRCIN1axis. Cancer Biomark. Sect. A Dis. Markers.

[104] Wang R.Q., Long X.R., Zhou N.N., Chen D.N., Zhang M.Y., Wen Z.S., Zhang L.J., He F.Z., Zhou Z.L., Mai S.J. (2021). Lnc-GAN1 expression is associated with good survival and suppresses tumor progression by sponging mir-26a-5p to activate PTEN signaling in non-small cell lung cancer. J. Exp. Clin. Cancer Res. CR.

[105] Gong Z., Chen X., Zhang Y., Liu C., Wang Z., Xu X., Zhu J., Xue T. (2020). LncRNA GATA6-AS1 Inhibits the Progression of Non-Small Cell Lung Cancer via Repressing microRNA-543 to Up-Regulating RKIP. Cancer Manag. Res..

[106] Ren K., Sun J., Liu L., Yang Y., Li H., Wang Z., Deng J., Hou M., Qiu J., Zhao W. (2021). TP53-Activated lncRNA GHRLOS Regulates Cell Proliferation, Invasion, and Apoptosis of Non-Small Cell Lung Cancer by Modulating the miR-346/APC Axis. Front. Oncol..

[107] Fan G., Jiao J., Shen F., Ren Q., Wang Q., Chu F. (2020). Long non-coding RNA HCG11 sponging miR-522-3p inhibits the tumorigenesis of non-small cell lung cancer by upregulating SOCS5. Thorac. Cancer.

[108] Tao X., Li Y., Fan S., Wu L., Xin J., Su Y., Xian X., Huang Y., Huang R., Fang W. (2023). Downregulation of Linc00173 increases BCL2 mRNA stability via the miR-1275/PROCA1/ZFP36L2 axis and induces acquired cisplatin resistance of lung adenocarcinoma. J. Exp. Clin. Cancer Res. CR.

[109] Ding D., Zhang J., Luo Z., Wu H., Lin Z., Liang W., Xue X. (2022). Analysis of the lncRNA-miRNA-mRNA Network Reveals a Potential Regulatory Mechanism of EGFR-TKI Resistance in NSCLC. Front. Genet..

[110] Dong J., Li B., Lin D., Lu D., Liu C., Lu X., Tang X., Li L., Zhu D., Liu J. (2020). LincRNA00494 Suppresses Non-small Cell Lung Cancer Cell Proliferation by Regulating SRCIN1 Expression as a ceRNA. Front. Oncol..

[111] Sui Y., Chi W., Feng L., Jiang J. (2020). LncRNA MAGI2-AS3 is downregulated in non-small cell lung cancer and may be a sponge of miR-25. BMC Pulm. Med..

[112] Ma J., Yan H., Zhang J., Tan Y., Gu W. (2019). Long-Chain Non-Coding RNA (lncRNA) MT1JP Suppresses Biological Activities of Lung Cancer by Regulating miRNA-423-3p/Bim Axis. Med. Sci. Monit. Int. Med. J. Exp. Clin. Res..

[113] Chen Z., Chen Z., Xu S., Zhang Q. (2021). LncRNA SOX2-OT/miR-30d-5p/PDK1 Regulates PD-L1 Checkpoint through the mTOR Signaling Pathway to Promote Non-small Cell Lung Cancer Progression and Immune Escape. Front. Genet..

[114] Xiao H., Liu Y., Liang P., Wang B., Tan H., Zhang Y., Gao X., Gao J. (2018). TP53TG1 enhances cisplatin sensitivity of non-small cell lung cancer cells through regulating miR-18a/PTEN axis. Cell Biosci..

[115] Cao F., Wang Z., Feng Y., Zhu H., Yang M., Zhang S., Wang X. (2020). lncRNA TPTEP1 competitively sponges miR-328-5p to inhibit the proliferation of non-small cell lung cancer cells. Oncol. Rep..

[116] Shi N., Feng D., Gu Y., Zheng C., Miao M. (2021). TUSC8 enhances cisplatin sensitivity of NSCLC cells through regulating VEGFA. J. BUON.

[117] Lin S., Zhang R., An X., Li Z., Fang C., Pan B., Chen W., Xu G., Han W. (2019). LncRNA HOXA-AS3 confers cisplatin resistance by interacting with HOXA3 in non-small-cell lung carcinoma cells. Oncogenesis.

[118] Wang J., Tian Y., Yu M., Ma M., Gao Y. (2023). Overexpression of the Long Noncoding RNA HIF2PUT Inhibits Non-Small Cell Lung Cancer Cell Proliferation and Invasion through HIF-2a Pathway. Cancer Biother. Radiopharm..

[119] Liang H., Peng J. (2022). LncRNA HOTAIR promotes proliferation, invasion and migration in NSCLC cells via the CCL22 signaling pathway. PLoS ONE.

[120] Gao Y.P., Li Y., Li H.J., Zhao B. (2019). LncRNA NBR2 inhibits EMT progression by regulating Notch1 pathway in NSCLC. Eur. Rev. Med. Pharmacol. Sci..

[121] Chang L., Xu W., Zhang Y., Gong F. (2019). Long non-coding RNA-NEF targets glucose transportation to inhibit the proliferation of non-small-cell lung cancer cells. Oncol. Lett..

[122] Huang J., Xie N., Huang H., Yao J., Hu W. (2019). Long noncoding RNA STXBP5-AS1 inhibits cell proliferation, migration, and invasion via preventing the PI3K/AKT against STXBP5 expression in non-small-cell lung carcinoma. J. Cell Biochem..

[123] Jiang X., Wang J., Fang L. (2020). LncRNA WT1-AS over-expression inhibits non-small cell lung cancer cell stemness by down-regulating TGF-beta1. BMC Pulm. Med..

[124] Tang L., Wang T., Zhang Y., Zhang J., Zhao H., Wang H., Wu Y., Liu K. (2019). Long Non-Coding RNA AWPPH Promotes Postoperative Distant Recurrence in Resected Non-Small Cell Lung Cancer by Upregulating Transforming Growth Factor beta 1 (TGF-beta1). Med. Sci. Monit. Int. Med. J. Exp. Clin. Res..

[125] Ju Z.S., Sun B., Bao D., Zhang X.F. (2020). Effect of lncRNA-BLACAT1 on drug resistance of non-small cell lung cancer cells in DDP chemotherapy by regulating cyclin D1 expression. Eur. Rev. Med. Pharmacol. Sci..

[126] Ding Z., Kang J., Yang Y. (2020). Long non-coding RNA CASC2 enhances irradiation-induced endoplasmic reticulum stress in NSCLC cells through PERK signaling. 3 Biotech.

[127] Liao J., Xie N. (2019). Long noncoding RNA DSCAM-AS1 functions as an oncogene in non-small cell lung cancer by targeting BCL11A. Eur. Rev. Med. Pharmacol. Sci..

[128] Gong W., Cao Y., Wang Y., Yang L., Su W., Qiu F., Datta S., Rao B., Xian J., Lin M. (2018). Upregulation of LncRNA FEZF-AS1 is associated with advanced clinical stages and family history of cancer in patients with NSCLC. Pathol. Res. Pract..

[129] Zheng F.X., Wang X.Q., Zheng W.X., Zhao J. (2019). Long noncoding RNA HOXA-AS2 promotes cell migration and invasion via upregulating IGF-2 in non-small cell lung cancer as an oncogene. Eur. Rev. Med. Pharmacol. Sci..

[130] Liu B., Li J., Li J.M., Liu G.Y., Wang Y.S. (2021). HOXC-AS2 mediates the proliferation, apoptosis, and migration of non-small cell lung cancer by combining with HOXC13 gene. Cell Cycle.

[131] Zhu H., Zhou Y., Wang Q., Yang X., Ding C., Xiong Y. (2021). Long non-coding RNA LALTOP promotes non-small cell lung cancer progression by stabilizing topoisomerase IIalpha mRNA. Biochem. Biophys. Res. Commun..

[132] Bian C., Yuan L., Gai H. (2019). A long non-coding RNA LINC01288 facilitates non-small cell lung cancer progression through stabilizing IL-6 mRNA. Biochem. Biophys. Res. Commun..

[133] Chen W., Zhao W., Chen S., Zhang L., Guo Z., Wang L., Wang J., Wan Z., Hong Y., Yu L. (2018). Expression and correlation of MALAT1 and SOX9 in non-small cell lung cancer. Clin. Respir. J..

[134] Wu Y., Shen Q.W., Niu Y.X., Chen X.Y., Liu H.W., Shen X.Y. (2020). LncNORAD interference inhibits tumor growth and lung cancer cell proliferation, invasion and migration by down-regulating CXCR4 to suppress RhoA/ROCK signaling pathway. Eur. Rev. Med. Pharmacol. Sci..

[135] Shen Q., Zhou H., Zhang M., Wu R., Wang L., Wang Y., Chen J. (2022). Super enhancer-LncRNA SENCR promoted cisplatin resistance and growth of NSCLC through upregulating FLI1. J. Clin. Lab. Anal..

[136] Zhu B., Finch-Edmondson M., Leong K.W., Zhang X., Mitheera V., Lin Q.X., Lee Y., Ng W.T., Guo H., Wan Y. (2021). LncRNA SFTA1P mediates positive feedback regulation of the Hippo-YAP/TAZ signaling pathway in non-small cell lung cancer. Cell Death Discov..

[137] Chen K., Abuduwufuer A., Zhang H., Luo L., Suotesiyali M., Zou Y. (2019). SNHG7 mediates cisplatin-resistance in non-small cell lung cancer by activating PI3K/AKT pathway. Eur. Rev. Med. Pharmacol. Sci..

[138] Qin Z., Zheng X., Fang Y. (2019). Long noncoding RNA TMPO-AS1 promotes progression of non-small cell lung cancer through regulating its natural antisense transcript TMPO. Biochem. Biophys. Res. Commun..

[139] Chen T., Li J., Zhou M.H., Xu L.J., Pan T.C. (2020). IL-6 stimulates lncRNA ZEB2-AS1 to aggravate the progression of non-small cell lung cancer through activating STAT1. Eur. Rev. Med. Pharmacol. Sci..

[140] He S., Lin J., Xu Y., Lin L., Feng J. (2019). A positive feedback loop between ZNF205-AS1 and EGR4 promotes non-small cell lung cancer growth. J. Cell Mol. Med..

[141] Mercer T.R., Dinger M.E., Mattick J.S. (2009). Long non-coding RNAs: Insights into functions. Nat. Rev. Genet..

[142] Ulitsky I. (2016). Evolution to the rescue: Using comparative genomics to understand long non-coding RNAs. Nat. Rev. Genet..

[143] Yang J., Qian X., Qiu Q., Xu L., Pan M., Li J., Ren J., Lu B., Qiu T., Chen E. (2022). LCAT1 is an oncogenic LncRNA by stabilizing the IGF2BP2-CDC6 axis. Cell Death Dis..

[144] Brownmiller T., Juric J.A., Ivey A.D., Harvey B.M., Westemeier E.S., Winters M.T., Stevens A.M., Stanley A.N., Hayes K.E., Sprowls S.A. (2020). Y Chromosome LncRNA Are Involved in Radiation Response of Male Non-Small Cell Lung Cancer Cells. Cancer Res..

[145] Zhu L., Liu Y., Tang H., Wang P. (2022). FOXP3 activated-LINC01232 accelerates the stemness of non-small cell lung carcinoma by activating TGF-beta signaling pathway and recruiting IGF2BP2 to stabilize TGFBR1. Exp. Cell Res..

[146] Han L., Lei G., Chen Z., Zhang Y., Huang C., Chen W. (2021). IGF2BP2 Regulates MALAT1 by Serving as an N6-Methyladenosine Reader to Promote NSCLC Proliferation. Front. Mol. Biosci..

[147] Zhu Q., Zhang C., Qu T., Lu X., He X., Li W., Yin D., Han L., Guo R., Zhang E. (2022). MNX1-AS1 Promotes Phase Separation of IGF2BP1 to Drive c-Myc-Mediated Cell-Cycle Progression and Proliferation in Lung Cancer. Cancer Res..

[148] Jiao P.F., Tang P.J., Chu D., Li Y.M., Xu W.H., Ren G.F. (2021). Long Non-Coding RNA THOR Depletion Inhibits Human Non-Small Cell Lung Cancer Cell Growth. Front. Oncol..

[149] Gong F., Dong D., Zhang T., Xu W. (2019). Long non-coding RNA FENDRR attenuates the stemness of non-small cell lung cancer cells via decreasing multidrug resistance gene 1 (MDR1) expression through competitively binding with RNA binding protein HuR. Eur. J. Pharmacol..

[150] Shan K.Z., Yang S.F., Deng Y.J., Yue P.Y., Du Z.Q. (2022). E2F1-induced long non-coding RNA MCF2L-AS1 modulates Cyclin D1 mRNA stability through ELAVL1 to induce Gefitinib resistance in non-small cell lung cancer. Acta Biochim. Pol..

[151] Huang Y., Xia L., Tan X., Zhang J., Zeng W., Tan B., Yu X., Fang W., Yang Z. (2022). Molecular mechanism of lncRNA SNHG12 in immune escape of non-small cell lung cancer through the HuR/PD-L1/USP8 axis. Cell Mol. Biol. Lett..

[152] Du Z., Niu S., Wang J., Wu J., Li S., Yi X. (2021). SChLAP1 contributes to non-small cell lung cancer cell progression and immune evasion through regulating the AUF1/PD-L1 axis. Autoimmunity.

[153] Zhang M., Wu J., Zhong W., Zhao Z., He W. (2021). DNA-methylation-induced silencing of DIO3OS drives non-small cell lung cancer progression via activating hnRNPK-MYC-CDC25A axis. Mol. Ther. Oncolytics.

[154] Pan J., Tang Y., Liu S., Li L., Yu B., Lu Y., Wang Y. (2020). LIMD1-AS1 suppressed non-small cell lung cancer progression through stabilizing LIMD1 mRNA via hnRNP U. Cancer Med..

[155] Wang W., Zhao Z., Xu C., Li C., Ding C., Chen J., Chen T., Zhao J. (2021). LncRNA FAM83A-AS1 promotes lung adenocarcinoma progression by enhancing the pre-mRNA stability of FAM83A. Thorac. Cancer.

[156] Yang H., Yang W., Dai W., Ma Y., Zhang G. (2020). LINC00667 promotes the proliferation, migration, and pathological angiogenesis in non-small cell lung cancer through stabilizing VEGFA by EIF4A3. Cell Biol. Int..

[157] Wang X., Yu X., Wei W., Liu Y. (2020). Long noncoding RNA MACC1-AS1 promotes the stemness of nonsmall cell lung cancer cells through promoting UPF1-mediated destabilization of LATS1/2. Environ. Toxicol..

[158] Luo M., Xie L., Su Y., Zhang K., Liang R., Ma Z., Li Y. (2022). TM4SF19-AS1 facilitates the proliferation of lung squamous cell carcinoma by recruiting WDR5 to mediate TM4SF19. Mol. Cell Probes.

[159] Zhang Q., Zhang Y., Chen H., Sun L.N., Zhang B., Yue D.S., Wang C.L., Zhang Z.F. (2022). METTL3-induced DLGAP1-AS2 promotes non-small cell lung cancer tumorigenesis through m^6^A/c-Myc-dependent aerobic glycolysis. Cell Cycle.

[160] Meyer K.D., Jaffrey S.R. (2014). The dynamic epitranscriptome: N6-methyladenosine and gene expression control. Nat. Rev. Mol. Cell Biol..

[161] Zaccara S., Ries R.J., Jaffrey S.R. (2019). Reading, writing and erasing mRNA methylation. Nat. Rev. Mol. Cell Biol..

[162] Brennan C.M., Steitz J.A. (2001). HuR and mRNA stability. Cell Mol. Life Sci. CMLS.

[163] Lebedeva S., Jens M., Theil K., Schwanhausser B., Selbach M., Landthaler M., Rajewsky N. (2011). Transcriptome-wide analysis of regulatory interactions of the RNA-binding protein HuR. Mol. Cell.

[164] Geuens T., Bouhy D., Timmerman V. (2016). The hnRNP family: Insights into their role in health and disease. Hum. Genet..

[165] Yang Y.W., Flynn R.A., Chen Y., Qu K., Wan B., Wang K.C., Lei M., Chang H.Y. (2014). Essential role of lncRNA binding for WDR5 maintenance of active chromatin and embryonic stem cell pluripotency. eLife.

[166] Zhu Y., Ren C., Yang L. (2021). Effect of eukaryotic translation initiation factor 4A3 in malignant tumors. Oncol. Lett..

[167] Sakellariou D., Frankel L.B. (2021). EIF4A3: A gatekeeper of autophagy. Autophagy.

[168] Tang Y., Feinberg T., Keller E.T., Li X.Y., Weiss S.J. (2016). Snail/Slug binding interactions with YAP/TAZ control skeletal stem cell self-renewal and differentiation. Nat. Cell Biol..

[169] Wang L., Zhang Z., Yu X., Huang X., Liu Z., Chai Y., Yang L., Wang Q., Li M., Zhao J. (2019). Unbalanced YAP-SOX9 circuit drives stemness and malignant progression in esophageal squamous cell carcinoma. Oncogene.

[170] Patil D.P., Pickering B.F., Jaffrey S.R. (2018). Reading m(6)A in the Transcriptome: M(6)A-Binding Proteins. Trends Cell Biol..

[171] Guttman M., Donaghey J., Carey B.W., Garber M., Grenier J.K., Munson G., Young G., Lucas A.B., Ach R., Bruhn L. (2011). lincRNAs act in the circuitry controlling pluripotency and differentiation. Nature.

[172] Herbst R.S., Morgensztern D., Boshoff C. (2018). The biology and management of non-small cell lung cancer. Nature.

[173] Kakumani P.K. (2022). AGO-RBP crosstalk on target mRNAs: Implications in miRNA-guided gene silencing and cancer. Transl. Oncol..

[174] Huang H., Li L., Wen K. (2021). Interactions between long non-coding RNAs and RNA-binding proteins in cancer. Oncol. Rep..

[175] Yao Z.T., Yang Y.M., Sun M.M., He Y., Liao L., Chen K.S., Li B. (2022). New insights into the interplay between long non-coding RNAs and RNA-binding proteins in cancer. Cancer Commun..

